# Some anomalous exact solutions for the four-component coupled nonlinear Schrödinger equations on complex wave backgrounds

**DOI:** 10.1038/s41598-022-20253-0

**Published:** 2022-09-30

**Authors:** Lu Wang, Li Li, Fajun Yu

**Affiliations:** School of Mathematics and Systematic Sciences, Shenyang Normal University, Shenyang, 110034 China

**Keywords:** Mathematics and computing, Applied mathematics, Computational science

## Abstract

The coupled nonlinear Schrödinger (CNLS) equations are the important models for the study of the multicomponent Bose-Einstein condensates (BECs). In this paper, we study the four-components CNLS equations via Darboux transformation and obtain the *N*-soliton solutions with zero seed and non-zero seed solutions($$q_i=0$$ or $$q_i=e^{-2it})$$. The 1-soliton solution and 2-soliton solution are calculated on complex wave backgrounds, the dark-bright-bright-bright soliton solutions and dark-dark-bright-bright soliton solutions are constructed. We can obtain a new class of dark-bright-bright-bright soliton solutions, which admit one-valley dark soliton in component $$q_1$$ and triple-hump bright solitons in the other three components. The collision properties between dark-dark-bright-bright solitons are considered, and the vector solitons are expected to be much more abundant than those of previously reported vector soliton collisions.

## Introduction

As early as 1920, the scientists Bose and Einstein believed that all quantum states of atoms would be clustered in a single quantum state based on Bose’s statistical mechanics of photons, which attracted the attention of physicists. In the following decades, the abundant scientists began to explore Bose-Einstein condensation(BEC) through some experiments. For example, London suggested that the tidal phenomenon of liquid nitrogen could be the BEC of helium atoms at low temperatures^1^. Bogolyubov described the theoretical model of weakly interacting Bose gases^2^. Hulin suggested that BEC experiments could be performed with exciton in cuprous oxide^3^. It was not until 1995 that the first BEC was obtained in experiments of the gaseous rubidium atoms by Keightley, Cornell and Wiman^4^. This was an important breakthrough in Bose-Einstein condensation experiments. Initially, these experiments were performed with single atom, meaning that most of the Bose gases occupied the same quantum state, called single-component BEC. With the development of science, the quantum states of BEC experiments were no longer limited to one quantum state, so multicomponent BECs were further studied. The simplest multicomponent BECs are two-component BECs. In this paper, we focus on the model of the four-component BECs and their exact solutions.

The nonlinear partial differential equation is an important tool in modern mathematics, such as KdV equation^5^:

$$\begin{aligned} q_t+6qq_x+q_{xxx}=0, \end{aligned}$$

the Gross-Pitaevskii (GP) equation^6–8^:

$$\begin{aligned} i\hbar u_t=-\frac{h^2}{2m}\nabla ^2u+{\overline{V}}_d(x)u+\frac{4\pi \hbar ^2a_s}{m}|u|^2u, x\in R^d,t\ge 0, \end{aligned}$$

and the nonlinear Schrödinger (NLS) equation^9^:

$$\begin{aligned} iq_t+q_{xx}\pm 2q^2q^*=0. \end{aligned}$$

Among them, the NLS equation is widely used in the study of nonlinear optics, Bose-Einstein condensation and other physics. For the multicomponent BECs, most of them are studied by the CNLS equations. Qin and other scholars gave the four-component BECs with repulsive interactions to establish the four-component CNLS equations^10^.

When theCNLS equations are given, it is particularly important work to solve their soliton solutions. The basic idea of Darboux transformation: It uses a seed solution of the nonlinear evolution equation and the solution of its Lax pairs, then obtains the new solution of the nonlinear evolution equation and the corresponding solution of its Lax pairs via the algebraic algorithm and differential operation^11,12^. Li and other researchers use Darboux transformation to solve the two-dimensional NLS equation with PT symmetry potential^13^. In addition, the NLS equation can be solved by Hirota bilinear method^14^, inverse scattering method^15^, Bäcklund transform method^16^, and so on. Subsequently, some researchers used Darboux transformation to find the exact solution of the CNLS equations. Priya et.al use the generalized Darboux transformation to obtain the N-order rogue wave solutions of the CNLS equations with cross-phase modulation and four-wave mixing terms^17–19^. Some soliton methods are proposed to solve the NLS equation and CNLS equations with a (space, time)-modulated external potential in Ref.^20–23^. The physical exact nonlinear solutions of the (n+1)-dimensional Schrödinger are obtained using RB sub-ODE and He’s semi-inverse techniques, and several types of solutions, bright optical and dark solitons are derived in^24^. Some extract new solutions of space-time stochastic fractional nonlinear Schrödinger equation with spatiotemporal dispersion are presented, and some new stochastic solutions with physical parameters are constructed via exponential distribution in^25^.

In this paper, we mainly use Darboux transform method to investigate the four-component CNLS equations, which is a tedious process compared with the two-component and three-component CNLS equations. By using the Lax pair already given in the literature, we construct the matrix Lax pairs in the spectral problem, then the the dark-bright-bright-bright soliton solutions and dark-dark-bright-bright soliton solutions of the four-component CNLS equations are calculated via the zero seed solution and the non-zero seed solution, the dynamical behaviors of the soliton solutions are shown on complex wave backgrounds.

## Results

### Darboux transformation for four-component CNLS equations

Since most of the CNLS equations are non-productible and contain imaginary numbers, the CNLS equations with some components are also more computationally intensive, so some traditional methods are not effective in solving this problem. In the following, with the help of the Lax pairs in the literature [9], the exact solutions of the four-component CNLS equations are calculated by constructing Darboux transformation, then the interactions between the solitary solutions are presented on complex wave backgrounds.

We consider the following four-component CNLS equations^9^:

1 $$\begin{aligned} \begin{array}{l} \left\{ \begin{array}{lllllllll} iq_{1,t}+\frac{1}{2}q_{1,xx}- \left( |q_1|^2+|q_2|^2+|q_3|^2+|q_4|^2 \right) q_1=0,\\ iq_{2,t}+\frac{1}{2}q_{2,xx}-\left( |q_1|^2+|q_2|^2+|q_3|^2+|q_4|^2 \right) q_2=0,\\ iq_{3,t}+\frac{1}{2}q_{3,xx}-\left( |q_1|^2+|q_2|^2+|q_3|^2+|q_4|^2 \right) q_3=0,\\ iq_{4,t}+\frac{1}{2}q_{4,xx}-\left( |q_1|^2+|q_2|^2+|q_3|^2+|q_4|^2 \right) q_4=0, \end{array}\right. \end{array} \end{aligned}$$

the multicomponent BECs provide a good platform for the investigation of vector solitons both theoretically and experimentally due to the abundance of intra- and interatomic interactions. And many more dark vector solitons have been experimentally observed in multicomponent repulsive BECs, the experimental observations of the collisions of bright-dark-bright solitons are realized in three-component BECs with repulsive interactions^26^.

The Lax pairs of Eq. (1) take the following forms:

2 $$\begin{aligned} \begin{array}{l} \psi _x=U(\lambda ;Q)\psi = \left( i\lambda \sigma _3+iQ \right) \psi =\left( \begin{array}{llllllll} i\lambda &{}-iq^*_1&{}-iq^*_2&{}-iq^*_3&{}-iq^*_4\\ iq_1&{}-i\lambda &{}0&{}0&{}0\\ iq_2&{}0&{}-i\lambda &{}0&{}0\\ iq_3&{}0&{}0&{}-i\lambda &{}0\\ iq_4&{}0&{}0&{}0&{}-i\lambda \end{array}\right) {\psi }, \end{array} \end{aligned}$$

where

$$\begin{aligned} \sigma _3=\left( \begin{array}{llllllll}1 &{}0&{}0&{}0&{}0\\ 0&{}-1&{}0&{}0&{}0\\ 0&{}0&{}-1&{}0&{}0\\ 0&{}0&{}0&{}-1&{}0\\ 0&{}0&{}0&{}0&{}-1 \end{array}\right) ,Q=\left( \begin{array}{llllllll} 0 &{}-q^*_1&{}-q^*_2&{}-q^*_3&{}-q^*_4\\ q_1&{}0&{}0&{}0&{}0\\ q_2&{}0&{}0&{}0&{}0\\ q_3&{}0&{}0&{}0&{}0\\ q_4&{}0&{}0&{}0&{}0 \end{array}\right) , \end{aligned}$$

and

3 $$\begin{aligned} \begin{array}{l} \varphi _t=V(\lambda ;Q)\psi =[i\lambda ^2\sigma _3+i\lambda Q-\frac{1}{2}(i\sigma _3Q^2-\sigma _3Q_x)]\psi =\\ \left( \begin{array}{llllllll} i\lambda ^2+\frac{1}{2}i(q^*_1q_1+q^*_2q_2+q^*_3q_3+q^*_4q_4)&{}-i\lambda q^*_1-\frac{1}{2}q^*_{1,x}&{}-i\lambda q^*_2-\frac{1}{2}q^*_{2,x}&{}-i\lambda q^*_3-\frac{1}{2}q^*_{3,x}&{}-i\lambda q^*_4-\frac{1}{2}q^*_{4,x}\\ i\lambda q_1-\frac{1}{2}q_{1,x}&{}-i\lambda ^2-\frac{1}{2}iq_1q^*_1&{}-\frac{1}{2}iq_1q^*_2&{}-\frac{1}{2}iq_1q^*_3&{}-\frac{1}{2}iq_1q^*_4\\ i\lambda q_2-\frac{1}{2}q_{2,x}&{}-\frac{1}{2}iq_2q^*_1&{}-i\lambda ^2-\frac{1}{2}iq_2q^*_2&{}-\frac{1}{2}iq_2q^*_3&{}-\frac{1}{2}iq_2q^*_4\\ i\lambda q_3-\frac{1}{2}q_{3,x}&{}-\frac{1}{2}iq_3q^*_1&{}-\frac{1}{2}iq_3q^*_2&{}-i\lambda ^2-\frac{1}{2}iq_3q^*_3&{}-\frac{1}{2}iq_3q^*_4\\ i\lambda q_4-\frac{1}{2}q_{4,x}&{}-\frac{1}{2}iq_4q^*_1&{}-\frac{1}{2}iq_4q^*_2&{}-\frac{1}{2}iq_4q^*_3&{}-i\lambda ^2-\frac{1}{2}iq_4q^*_4 \end{array}\right) {\psi }. \end{array} \end{aligned}$$

Here $*$ is the complex conjugate, $q_i(i=1,2,3,4)$ is a function of the variable space *x* and time variable *t*, the $\lambda$ is the universal parameter.

Constructing Darboux transformation of Eq. (1) by introducing the transformation matrix *T*:

4 $$\begin{aligned} \begin{array}{l} {\widetilde{\psi }}=T\psi , T=\left( \begin{array}{llllllll}T_{11} &{}T_{12}&{}T_{13}&{}T_{14}&{}T_{15}\\ T_{21}&{}T_{22}&{}T_{23}&{}T_{24}&{}T_{25}\\ T_{31}&{}T_{32}&{}T_{33}&{}T_{34}&{}T_{35}\\ T_{41}&{}T_{42}&{}T_{43}&{}T_{44}&{}T_{45}\\ T_{51}&{}T_{52}&{}T_{53}&{}T_{54}&{}T_{55}\end{array}\right) . \end{array} \end{aligned}$$

Using the compatibility, we can yield:

5 $$\begin{aligned} \begin{array}{l} \psi _x={\widetilde{U}}\psi ,{\widetilde{U}}= \left( T_x+i\lambda T\sigma _3+iT{\widetilde{Q}}\right) T^{-1}, \end{array} \end{aligned}$$

and

6 $$\begin{aligned} \begin{array}{l} \psi _t={\widetilde{V}}\psi ,{\widetilde{V}}= \left( T_t+i\lambda ^2T\sigma _3+i\lambda T{\widetilde{Q}}-\frac{1}{2}iT\sigma _3{\widetilde{Q}}^{2}+\frac{1}{2}T\sigma _3\widetilde{Q_x}\right) T^{-1}. \end{array} \end{aligned}$$

If two Lax pairs ${\widetilde{U}}$, ${\widetilde{V}}$ and *U*, *V* have the same types in systems (5) and (6), the $\varphi =\left( \varphi _1, \varphi _2, \varphi _3, \varphi _4, \varphi _5 \right) ^T$, $\phi =\left( \phi _1,\phi _2, \phi _3,\phi _4, \phi _5 \right) ^T$, $\eta = \left( \eta _1, \eta _2, \eta _3, \eta _4, \eta _5 \right) ^T$,$\xi =\left( \xi _1, \xi _2,\xi _3, \xi _4, \xi _5 \right) ^T$, $\varepsilon = \left( \varepsilon _1, \varepsilon _2, \varepsilon _3, \varepsilon _4, \varepsilon _5 \right) ^T$ are the basic solutions of Eqs. (2) and (3), then we give the following linear algebraic systems:

7 $$\begin{aligned} \begin{array}{l} \left\{ \begin{array}{lllllllll} \sum _{i=0}^{N-1}\left( A_{11}^{(i)}+A_{12}^{(i)}M_j^{(1)}+A_{13}^{(i)}M_j^{(2)}+A_{14}^{(i)}M_j^{(3)}+A_{15}^{(i)}M_j^{(4)}\right) \lambda _j^{i}=-\lambda _j^{N},\\ \sum _{i=0}^{N-1}\left( A_{21}^{(i)}+A_{22}^{(i)}M_j^{(1)}+A_{23}^{(i)}M_j^{(2)}+A_{24}^{(i)}M_j^{(3)}+A_{25}^{(i)}M_j^{(4)}\right) \lambda _j^{i}=-M_j^{(1)}\lambda _j^{N},\\ \sum _{i=0}^{N-1}\left( A_{31}^{(i)}+A_{32}^{(i)}M_j^{(1)}+A_{33}^{(i)}M_j^{(2)}+A_{34}^{(i)}M_j^{(3)}+A_{35}^{(i)}M_j^{(4)}\right) \lambda _j^{i}=-M_j^{(2)}\lambda _j^{N},\\ \sum _{i=0}^{N-1}\left( A_{41}^{(i)}+A_{42}^{(i)}M_j^{(1)}+A_{43}^{(i)}M_j^{(2)}+A_{44}^{(i)}M_j^{(3)}+A_{45}^{(i)}M_j^{(4)}\right) \lambda _j^{i}=-M_j^{(3)}\lambda _j^{N},\\ \sum _{i=0}^{N-1}\left( A_{51}^{(i)}+A_{52}^{(i)}M_j^{(1)}+A_{53}^{(i)}M_j^{(2)}+A_{54}^{(i)}M_j^{(3)}+A_{55}^{(i)}M_j^{(4)}\right) \lambda _j^{i}=-M_j^{(4)}\lambda _j^{N}. \end{array}\right. \end{array} \end{aligned}$$

with

8 $$\begin{aligned} \begin{array}{l} M_j^{(1)}=\frac{\varphi _2+v^{(11)}_j\phi _2+v^{(12)}_j\eta _2+v^{(13)}_j\xi _2+v^{(14)}_j\varepsilon _2}{\varphi _1+v^{(11)}_j\phi _1+v^{(12)}_j\eta _1+v^{(13)}_j\xi _1+v^{(14)}_j\varepsilon _1},\\ M_j^{(2)}=\frac{\varphi _3+v^{(21)}_j\phi _3+v^{(22)}_j\eta _3+v^{(23)}_j\xi _3+v^{(24)}_j\varepsilon _3}{\varphi _1+v^{(21)}_j\phi _1+v^{(22)}_j\eta _1+v^{(23)}_j\xi _1+v^{(24)}_j\varepsilon _1},\\ M_j^{(3)}=\frac{\varphi _4+v^{(31)}_j\phi _4+v^{(32)}_j\eta _4+v^{(33)}_j\xi _4+v^{(34)}_j\varepsilon _4}{\varphi _1+v^{(31)}_j\phi _1+v^{(32)}_j\eta _1+v^{(33)}_j\xi _1+v^{(34)}_j\varepsilon _1},\\ M_j^{(4)}=\frac{\varphi _5+v^{(41)}_j\phi _5+v^{(42)}_j\eta _5+v^{(43)}_j\xi _5+v^{(44)}_j\varepsilon _5}{\varphi _1+v^{(41)}_j\phi _1+v^{(42)}_j\eta _1+v^{(43)}_j\xi _1+v^{(44)}_j\varepsilon _1},0\le j\le 5N, \end{array} \end{aligned}$$

here $\lambda _j$ and $v^{(k)}_j$ are given the right parameters, so that the Eqs. (7) and (8) are not zero. We consider a $5\times 5$ matrix *T*, and *T* is of the form as following:

9 $$\begin{aligned} \begin{array}{l} \left\{ \begin{array}{lllllllll} T_{11}=\lambda ^{N}+\sum _{i=0}^{N-1}A_{11}^{(i)}\lambda ^{i},T_{12}=\sum _{i=0}^{N-1}A_{12}^{(i)}\lambda ^{i},T_{13}=\sum _{i=0}^{N-1}A_{13}^{(i)}\lambda ^{i},T_{14}=\sum _{i=0}^{N-1}A_{14}^{(i)}\lambda ^{i},T_{15}=\sum _{i=0}^{N-1}A_{15}^{(i)}\lambda ^{i},\\ T_{21}=\sum _{i=0}^{N-1}A_{21}^{(i)}\lambda ^{i},T_{22}=\lambda ^{N}+\sum _{i=0}^{N-1}A_{22}^{(i)}\lambda ^{i},T_{23}=\sum _{i=0}^{N-1}A_{23}^{(i)}\lambda ^{i},T_{24}=\sum _{i=0}^{N-1}A_{24}^{(i)}\lambda ^{i},T_{25}=\sum _{i=0}^{N-1}A_{25}^{(i)}\lambda ^{i},\\ T_{31}=\sum _{i=0}^{N-1}A_{31}^{(i)}\lambda ^{i},T_{32}=\sum _{i=0}^{N-1}A_{32}^{(i)}\lambda ^{i},T_{33}=\lambda ^{N}+\sum _{i=0}^{N-1}A_{33}^{(i)}\lambda ^{i},T_{34}=\sum _{i=0}^{N-1}A_{34}^{(i)}\lambda ^{i},T_{35}=\sum _{i=0}^{N-1}A_{35}^{(i)}\lambda ^{i},\\ T_{41}=\sum _{i=0}^{N-1}A_{41}^{(i)}\lambda ^{i},T_{42}=\sum _{i=0}^{N-1}A_{42}^{(i)}\lambda ^{i},T_{43}=\sum _{i=0}^{N-1}A_{43}^{(i)}\lambda ^{i},T_{44}=\lambda ^{N}+\sum _{i=0}^{N-1}A_{22}^{(i)}\lambda ^{i},T_{45}=\sum _{i=0}^{N-1}A_{45}^{(i)}\lambda ^{i},\\ T_{51}=\sum _{i=0}^{N-1}A_{51}^{(i)}\lambda ^{i},T_{52}=\sum _{i=0}^{N-1}A_{52}^{(i)}\lambda ^{i},T_{53}=\sum _{i=0}^{N-1}A_{53}^{(i)}\lambda ^{i},T_{54}=\sum _{i=0}^{N-1}A_{54}^{(i)}\lambda ^{i},T_{55}=\lambda ^{N}+\sum _{i=0}^{N-1}A_{55}^{(i)}\lambda ^{i}, \end{array}\right. \end{array} \end{aligned}$$

where *N* is a natural number, the $A_{mn}^{(i)}(m,n=1,2,3,4,5. i\ge 0)$ are the functions of *x* and *t*. By calculations, we can obtain $\Delta T$ as following

10 $$\begin{aligned} \begin{array}{l} \Delta T=\prod _{j=1}^{5N}(\lambda -\lambda _j), \end{array} \end{aligned}$$

which proves that $\lambda _j$ $(j=1\le j\le 5N,)$ are 5*N* roots of $\Delta T$. Based on these above conditions, we will proof that ${\widetilde{U}}$ and ${\widetilde{V}}$ have the same forms with *U* and *V*, respectively.

#### Proposition 1

The matrix ${\widetilde{U}}$ defined by Eq. (5) has the same type as *U*, we have

11 $$\begin{aligned} \begin{array}{l}{\widetilde{U}}= \left( \begin{array}{llllllll} i\lambda &{}-i{\widetilde{q}}^*_1&{}-i{\widetilde{q}}^*_2&{}-i{\widetilde{q}}^*_3&{}-i{\widetilde{q}}^*_4\\ i{\widetilde{q}}_1&{}-i\lambda &{}0&{}0&{}0\\ i{\widetilde{q}}_2&{}0&{}-i\lambda &{}0&{}0\\ i{\widetilde{q}}_3&{}0&{}0&{}-i\lambda &{}0\\ i{\widetilde{q}}_4&{}0&{}0&{}0&{}-i\lambda \end{array}\right) , \end{array} \end{aligned}$$

the relations between the new solution and the old solution of Eq. (5) are given:

12 $$\begin{aligned} \begin{array}{l} \left\{ \begin{array}{lllllllll} {\widetilde{q}}_1=q_1+2A^{(N-1)}_{21},\\ {\widetilde{q}}_2=q_2+2A^{(N-1)}_{31},\\ {\widetilde{q}}_3=q_3+2A^{(N-1)}_{41},\\ {\widetilde{q}}_4=q_4+2A^{(N-1)}_{51}. \end{array}\right. \end{array} \end{aligned}$$

#### Proof

Setting $$T^{-1}=\frac{T^*}{\Delta T}$$ and

13 $$\begin{aligned} \begin{array}{l} (T_x+TA)T^*=\left( \begin{array}{llllllll}B_{11}(\lambda ) &{}B_{12}(\lambda )&{}B_{13}(\lambda )&{}B_{14}(\lambda )&{}B_{15}(\lambda )\\ B_{21}(\lambda )&{}B_{22}(\lambda )&{}B_{23}(\lambda )&{}B_{24}(\lambda )&{}B_{25}(\lambda )\\ B_{31}(\lambda )&{}B_{32}(\lambda )&{}B_{33}(\lambda )&{}B_{34}(\lambda )&{}B_{35}(\lambda )\\ B_{41}(\lambda )&{}B_{42}(\lambda )&{}B_{43}(\lambda )&{}B_{44}(\lambda )&{}B_{45}(\lambda )\\ B_{51}(\lambda )&{}B_{52}(\lambda )&{}B_{53}(\lambda )&{}B_{54}(\lambda )&{}B_{55}(\lambda ) \end{array}\right) , \end{array} \end{aligned}$$

it is easy to verify that $B_{sl}$ $(1\le s,l\le 5)$ are 5*N* or $5N+1$ degree polynomials of $\lambda$. Through calculation, Eq. (13) can be obtained in the following form:

14 $$\begin{aligned} \begin{array}{l} (T_x+TU)T^*=(\Delta T)P(\lambda ), \end{array} \end{aligned}$$

where

15 $$\begin{aligned} \begin{array}{l} P(\lambda )=\left( \begin{array}{llllllll}P_{11}^{(1)}\lambda +P_{11}^{(0)} &{}P_{12}^{(0)}&{}P_{13}^{(0)}&{}P_{14}^{(0)}&{}P_{15}^{(0)}\\ P_{21}^{(0)}&{}P_{22}^{(1)}\lambda +P_{22}^{(0)}&{}P_{23}^{(0)}&{}P_{24}^{(0)}&{}P_{25}^{(0)}\\ P_{31}^{(0)}&{}P_{32}^{(0)}&{}P_{33}^{(1)}\lambda +P_{33}^{(0)}&{}P_{34}^{(0)}&{}P_{35}^{(0)}\\ P_{41}^{(0)}&{}P_{42}^{(0)}&{}P_{43}^{(0)}&{}P_{44}^{(1)}\lambda +P_{44}^{(0)}&{}P_{45}^{(0)}\\ P_{51}^{(0)}&{}P_{52}^{(0)}&{}P_{53}^{(0)}&{}P_{54}^{(0)}&{}P_{55}^{(1)}\lambda +P_{55}^{(0)} \end{array}\right) , \end{array} \end{aligned}$$

and $P_{mn}^{(k)} (m,n=1,2,3,4,5, k=0,1)$ satisfy the functions without $\lambda$. Based on Eq. (14), the system is obtained as following

16 $$\begin{aligned} \begin{array}{l} (T_x+TU)=P(\lambda )T. \end{array} \end{aligned}$$

Through comparing the $N+1, N, N-1$-th powers of $\lambda$ in Eq. (16), we have the following systems:

17 $$\begin{aligned} \begin{array}{l} \left\{ \begin{array}{lllllllll} P_{11}^{(1)}=i,P_{11}^{(0)}=0,P_{12}^{(0)}=-iq^*_1-2iA^{(N-1)}_{12},P_{13}^{(0)}=-iq^*_2-2iA^{(N-1)}_{13},\\ P_{14}^{(0)}=-iq^*_3-2iA^{(N-1)}_{14},P_{15}^{(0)}=-iq^*_4-2iA^{(N-1)}_5,\\ P_{21}^{(0)}=iq_1+2iA^{(N-1)}_{21},P_{22}^{(1)}=-i,P_{22}^{(0)}=0,P_{23}^{(0)}=0,P_{24}^{(0)}=0,P_{25}^{(0)}=0,\\ P_{31}^{(0)}=iq_2+2iA^{(N-1)}_{31},P_{32}^{(0)}=0,P_{33}^{(1)}=-i,P_{33}^{(0)}=0,P_{34}^{(0)}=0,P_{35}^{(0)}=0,\\ P_{41}^{(0)}=iq_3+2iA^{(N-1)}_{41},P_{42}^{(0)}=0,P_{43}^{(0)}=0,P_{44}^{(1)}=-i,P_{44}^{(0)}=0,P_{45}^{(0)}=0,\\ P_{51}^{(0)}=iq_4+2iA^{(N-1)}_{51},P_{52}^{(0)}=0,P_{53}^{(0)}=0,P_{54}^{(0)}=0,P_{55}^{(1)}=-i,P_{55}^{(0)}=0. \end{array}\right. \end{array} \end{aligned}$$

In this section, we set that the new matrix ${\widetilde{U}}$ has same type with *U*, which means that they have the same structures only $q, q^*$ of *U* transformed into ${{\widetilde{q}}}, {{\widetilde{q}}}^*$ of ${\widetilde{U}}$. After detailed calculation, we compare the ranks of $\lambda ^{N}$, and get the objective equations as following:

18 $$\begin{aligned} \begin{array}{l} \left\{ \begin{array}{lllllllll} {\widetilde{q}}_1=q_1+2A^{(N-1)}_{21},\\ {\widetilde{q}}_2=q_2+2A^{(N-1)}_{31},\\ {\widetilde{q}}_3=q_3+2A^{(N-1)}_{41},\\ {\widetilde{q}}_4=q_4+2A^{(N-1)}_{51}, \end{array}\right. \end{array} \end{aligned}$$

from Eqs. (17) and (18), we know that $\widetilde{U}=P(\lambda )$. The proof is completed. $\square$

#### Proposition 2

Under the transformation (6), the matrix ${\widetilde{V}}$ defined by Eq. (6) has the same form as *V*, that is,

19 $$\begin{aligned} \begin{array}{l} {\widetilde{V}}=\left( \begin{array}{llllllll}-i\lambda ^2+\frac{1}{2}i\tau &{} -i\lambda {\widetilde{q}}^*_1-\frac{1}{2}{\widetilde{q}}^*_{1,x}&{}-i\lambda {\widetilde{q}}^*_2-\frac{1}{2}{\widetilde{q}}^*_{2,x}&{}-i\lambda {\widetilde{q}}^*_3-\frac{1}{2}{\widetilde{q}}^*_{3,x}&{}-i\lambda {\widetilde{q}}^*_4-\frac{1}{2}{\widetilde{q}}^*_{4,x}\\ i\lambda {\widetilde{q}}_1-\frac{1}{2}{\widetilde{q}}_{1,x}&{}-i\lambda ^2-\frac{1}{2}i{\widetilde{q}}_1{\widetilde{q}}^*_1&{}-\frac{1}{2}i{\widetilde{q}}_1{\widetilde{q}}^*_2&{}-\frac{1}{2}i{\widetilde{q}}_1{\widetilde{q}}^*_3&{}-\frac{1}{2}i{\widetilde{q}}_1{\widetilde{q}}^*_4\\ i\lambda {\widetilde{q}}_2-\frac{1}{2}{\widetilde{q}}_{2,x}&{}-\frac{1}{2}i{\widetilde{q}}_2{\widetilde{q}}^*_1&{}-i\lambda ^2-\frac{1}{2}i{\widetilde{q}}_2{\widetilde{q}}^*_2&{}-\frac{1}{2}i{\widetilde{q}}_2{\widetilde{q}}^*_3&{}-\frac{1}{2}i{\widetilde{q}}_2{\widetilde{q}}^*_4\\ i\lambda {\widetilde{q}}_3-\frac{1}{2}{\widetilde{q}}_{3,x}&{}-\frac{1}{2}i{\widetilde{q}}_3{\widetilde{q}}^*_1&{}-\frac{1}{2}i{\widetilde{q}}_3{\widetilde{q}}^*_2&{}-i\lambda ^2-\frac{1}{2}i{\widetilde{q}}_3{\widetilde{q}}^*_3&{}-\frac{1}{2}i{\widetilde{q}}_3{\widetilde{q}}^*_4\\ i\lambda {\widetilde{q}}_4-\frac{1}{2}{\widetilde{q}}_{4,x}&{}-\frac{1}{2}i{\widetilde{q}}_4{\widetilde{q}}^*_1&{}-\frac{1}{2}i{\widetilde{q}}_4{\widetilde{q}}^*_2&{}-\frac{1}{2}i{\widetilde{q}}_4{\widetilde{q}}^*_3&{}-i\lambda ^2-\frac{1}{2}i{\widetilde{q}}_4{\widetilde{q}}^*_4 \end{array}\right) , \end{array} \end{aligned}$$

where $\tau ={\widetilde{q}}^*_1{\widetilde{q}}_1+{\widetilde{q}}^*_2{\widetilde{q}}_2+{\widetilde{q}}^*_3{\widetilde{q}}_3+{\widetilde{q}}^*_4{\widetilde{q}}_4,$ the relations between the new solutions and the old solutions of Eq. (6) are given:

20 $$\begin{aligned} \begin{array}{l} \left\{ \begin{array}{lllllllll} {\widetilde{q}}_1=q_1+2A^{(N-1)}_{21},\\ {\widetilde{q}}_2=q_2+2A^{(N-1)}_{31},\\ {\widetilde{q}}_3=q_3+2A^{(N-1)}_{41},\\ {\widetilde{q}}_4=q_4+2A^{(N-1)}_{51}. \end{array}\right. \end{array} \end{aligned}$$

#### Proof

Setting $$T^{-1}=\frac{T^*}{\Delta T}$$ and

21 $$\begin{aligned} \begin{array}{l} (T_t+TV)T^*=\left( \begin{array}{llllllll}C_{11}(\lambda )&{}C_{12}(\lambda )&{}C_{13}(\lambda )&{}C_{14}(\lambda )&{}C_{15}(\lambda )\\ C_{21}(\lambda )&{}C_{22}(\lambda )&{}C_{23}(\lambda )&{}C_{24}(\lambda )&{}C_{25}(\lambda )\\ C_{31}(\lambda )&{}C_{32}(\lambda )&{}C_{33}(\lambda )&{}C_{34}(\lambda )&{}C_{35}(\lambda )\\ C_{41}(\lambda )&{}C_{42}(\lambda )&{}C_{43}(\lambda )&{}C_{44}(\lambda )&{}C_{45}(\lambda )\\ C_{51}(\lambda )&{}C_{52}(\lambda )&{}C_{53}(\lambda )&{}C_{54}(\lambda )&{}C_{55}(\lambda )\end{array}\right) . \end{array} \end{aligned}$$

It is easy to verify that $C_{sl}$ $(1\le s,l\le 5)$ are 5*N* or $5N+1$ degree polynomials of $\lambda$.

Through some calculations, the $\lambda _j$ $(j=1\le j\le 5,)$ are the roots of $C_{sl}$ $(1\le s,l\le 5)$. Thus, Eq. (21) has the following structure

22 $$\begin{aligned} \begin{array}{l} (T_t+TV)T^*=(\Delta T)Q(\lambda ), \end{array} \end{aligned}$$

where

23 $$\begin{aligned} \begin{array}{l} Q(\lambda )=\left( \begin{array}{llllllll}\tau _{1}&\quad{}Q_{12}^{(1)}\lambda +Q_{12}^{(0)}&\quad{}Q_{13}^{(1)}\lambda +Q_{13}^{(0)}&\quad{}Q_{14}^{(1)}\lambda +Q_{14}^{(0)}&\quad{}Q_{15}^{(1)}\lambda +Q_{15}^{(0)}\\ Q_{21}^{(1)}\lambda +Q_{21}^{(0)}&\quad{}\tau _{2}&\quad{}Q_{23}^{(1)}\lambda +Q_{23}^{(0)}&\quad{}Q_{24}^{(1)}\lambda +Q_{24}^{(0)}&\quad{}Q_{25}^{(1)}\lambda +Q_{25}^{(0)}\\ Q_{31}^{(1)}\lambda +Q_{31}^{(0)}&\quad{}Q_{32}^{(1)}\lambda +Q_{32}^{(0)}&\quad{}\tau _{3}&\quad{}Q_{34}^{(1)}\lambda +Q_{34}^{(0)}&\quad{}Q_{35}^{(1)}\lambda +Q_{35}^{(0)}\\ Q_{41}^{(1)}\lambda +Q_{41}^{(0)}&\quad{}Q_{42}^{(1)}\lambda +Q_{42}^{(0)}&\quad{}Q_{43}^{(1)}\lambda +Q_{43}^{(0)}&\quad{}\tau _{4}&\quad{}Q_{45}^{(1)}\lambda +Q_{45}^{(0)}\\ Q_{51}^{(1)}\lambda +Q_{51}^{(0)}&\quad{}Q_{52}^{(1)}\lambda +Q_{52}^{(0)}&\quad{}Q_{53}^{(1)}\lambda +Q_{53}^{(0)}&\quad{}Q_{54}^{(1)}\lambda +Q_{54}^{(0)}&\quad{}\tau _{5} \end{array}\right) , \end{array} \end{aligned}$$

with $\tau _{1}=Q_{11}^{(2)}\lambda ^2+Q^{(1)}_{11}\lambda +Q^{(0)}_{11}, \tau _{2}=Q_{22}^{(2)}\lambda ^2+Q_{22}^{(1)}\lambda +Q_{22}^{(0)}, \tau _{3}=Q^{(2)}_{33}\lambda ^2+Q_{33}^{(1)}\lambda +Q_{33}^{(0)}, \tau _{4}=Q^{(2)}_{44}\lambda ^2+Q_{44}^{(1)}\lambda +Q_{44}^{(0)}, \tau _{5}=Q^{(2)}_{55}\lambda ^2+Q_{55}^{(1)}\lambda +Q_{55}^{(0)},$ and $Q_{mn}^{(k)} (m,n=1,2,3,4,5,k=0,1,2)$ satisfy the functions without $\lambda$. We obtain the following system from Eq. (22)

24 $$\begin{aligned} \begin{array}{l} T_t+TV=Q(\lambda )T. \end{array} \end{aligned}$$

By comparing the $N+2$, $N+1$, *N*-th powers of $\lambda$ in Eq. (24), we have the following system

25 $$\begin{aligned} \begin{array}{l} \left\{ \begin{array}{lllllllll} Q^{(2)}_{11}=i, Q^{(1)}_{11}=0, Q^{(0)}_{11}=\frac{1}{2}i({\tilde{q}}^*_1{\tilde{q}}_1+{\tilde{q}}^*_2{\tilde{q}}_2+{\tilde{q}}^*_3{\tilde{q}}_3+{\tilde{q}}^*_1=4{\tilde{q}}_4), Q^{(1)}_{1k}=-iq^*_{k-1}-2iA^{(N-1)}_{1k},\\ Q^{(0)}_{1k}=-\frac{1}{2}q^*_{k-1,x}-A^{(N-1)}_{1k,x}+2iA^{(N-2)}_{1k}, Q^{(1)}_{k1}=iq_{k-1}+2iA^{(N-1)}_{k1},\\ Q^{(0)}_{k1}=-\frac{1}{2}q_{k-1,x}-A^{(N-1)}_{k1,x}-2iA^{(N-2)}_{k1}, Q^{(2)}_{22}=Q^{(2)}_{33}=Q^{(2)}_{44}=Q^{(2)}_{55}=-i,\\ Q^{(1)}_{kk}=0, Q^{(0)}_{hh}=-\frac{1}{2}iq_{h-1}q^*_{h-1}-iq^*_{h-1}A^{(N-1)}_{h1}-iq_{h-1}A^{(N-1)}_{1h}-2iA^{(N-1)}_{h1}A^{(N-1)}_{1h},\\ Q^{(0)}_{kh}=-\frac{1}{2}iq_{k-1}q^*_{h-1}-iq^*_{h-1}A^{(N-1)}_{k1}-iq_{k-1}A^{(N-1)}_{1h}-2iA^{(N-1)}_{k1}A^{(N-1)}_{1h},\\ Q^{(0)}_{hk}=-\frac{1}{2}iq_{h-1}q^*_{k-1}-iq^*_{k-1}A^{(N-1)}_{h1}-iq_{h-1}A^{(N-1)}_{1k}-2iA^{(N-1)}_{h1}A^{(N-1)}_{1k}, \end{array}\right. \end{array} \end{aligned}$$

where $k=2,3,4,5$, $h=2,3,4,5,$ and $h=h$, *kh* and *hk* are referring to $k<h$.

In the section, we set that the new matrix ${\widetilde{V}}$ has same type with *V*, which means that they have the same structures only $q,q^*$ of *V* transformed into ${{\widetilde{q}}},{{\widetilde{q}}}^*$ of ${\widetilde{V}}$. After detailed calculations, we compare the ranks of $\lambda ^{N}$, and get the objective equations as following:

26 $$\begin{aligned} \begin{array}{l} \left\{ \begin{array}{lllllllll} {\widetilde{q}}_1=q_1+2A^{(N-1)}_{21},\\ {\widetilde{q}}_2=q_2+2A^{(N-1)}_{31},\\ {\widetilde{q}}_3=q_3+2A^{(N-1)}_{41},\\ {\widetilde{q}}_4=q_4+2A^{(N-1)}_{51}, \end{array}\right. \end{array} \end{aligned}$$

according to Eqs. (25) and (26), we know that $\widetilde{V}=Q(\lambda )$. The proof is completed. $\square$

## Conclusion and discussion

In this paper, the four-component CNLS equations are investigated, which describe the wave propagations of multicomponent Bose-Einstein condensates. Starting from its Lax pair with initial non-zero plane-wave conditions, we find the vector soliton solutions (non-zero backgrounds) and soliton-like solutions (zero backgrounds) with the aid of the modified Darboux transformation. Then the N-soliton solutions of the four-component CNLS equations in the case of zero-seeded and non-zero-seeded solutions are considered. And we construct three kinds of solutions including bright-bright-bright-bright soliton solutions, dark-bright-bright-bright soliton solutions and dark-dark -bright-bright soliton solutions. The 1-soliton solution and 2-soliton solution of the four-component CNLS equation are calculated using the zero seed solution and non-zero seed solution, the dynamical behaviors of the soliton solutions are shown on complex wave backgrounds.

Very recently, the non-degenerate bright solitons are discussed in the *N*-component coupled systems with attractive interactions based Hirota bilinear method. Our method can also be extended to *N*-component coupled systems with repulsive interactions. Moreover, some numerical simulations may show that these soliton states are robust against small deviations and weak noises. Therefore, there are many possibilities to observe them in real experiments.

## Methods

### N-soliton solutions for the four-component coupled NLS equation on complex wave backgrounds

#### N-soliton solutions on zero wave backgrounds

In order to obtain the *N*-soliton solution formula of four-component CNLS equations on zero wave backgrounds with Darboux transformation, we firstly give a set of seed solutions $q_1=q_2=q_3=q_4=0$ and substitute these solutions into Eqs. (4) and (5), which can get five basic solutions for CNLS equations:

27 $$\begin{aligned} \begin{array}{l} \varphi =\left( \begin{array}{llllllll}e^{i\lambda x+i\lambda ^2t} \\ 0\\ 0\\ 0\\ 0\end{array}\right) , \phi =\left( \begin{array}{llllllll}0\\ e^{-i\lambda x-i\lambda ^2t} \\ 0\\ 0\\ 0\end{array}\right) , \eta =\left( \begin{array}{llllllll}0\\ 0\\ e^{-i\lambda x-i\lambda ^2t} \\ 0\\ 0\end{array}\right) , \xi =\left( \begin{array}{llllllll}0\\ 0\\ 0\\ e^{-i\lambda x-i\lambda ^2t} \\ 0\end{array}\right) , \varepsilon =\left( \begin{array}{llllllll}0\\ 0\\ 0\\ 0\\ e^{-i\lambda x-i\lambda ^2t}\end{array}\right) . \end{array} \end{aligned}$$

Substituting Eq. (27) into Eq. (8), we give rise to

28 $$\begin{aligned} \begin{array}{l} M_j^{(1)}=e^{-2 \left( i\lambda x+i\lambda ^2t+F_{1,j}\right) }, M_j^{(2)}=e^{-2 \left( i\lambda x+i\lambda ^2t+F_{2,j}\right) },\\ M_j^{(3)}=e^{-2 \left( i\lambda x+i\lambda ^2t+F_{3,j}\right) }, M_j^{(4)}=e^{-2 \left( i\lambda x+i\lambda ^2t+F_{4,j}\right) }, \end{array} \end{aligned}$$

with $$e^{(-2F_{1,j})}=v^{(11)}_j$$, $$e^{(-2F_{2,j})}=v^{(22)}_j$$, $$e^{(-2F_{3,j})}=v^{(33)}_j$$, $$e^{(-2F_{4,j})}=v^{(44)}_j$$.

In order to obtain *N*-soliton solutions of Eq. (1) with zero wave backgrounds, we consider matrix *T* as following:

29 $$\begin{aligned} \begin{array}{l} T=\left( \begin{array}{llllllll}\lambda ^{N}+\sum ^{N-1}_{i=0}A_{11}\lambda ^i_j&{}\sum ^{N-1}_{i=0}A_{12}\lambda ^i_j&{}\sum ^{N-1}_{i=0}A_{13}\lambda ^i_j&{}\sum ^{N-1}_{i=0}A_{14}\lambda ^i_j&{}\sum ^{N-1}_{i=0}A_{15}\lambda ^i_j\\ \sum ^{N-1}_{i=0}A_{21}\lambda ^i_j&{}\lambda ^{N}+\sum ^{N-1}_{i=0}A_{22}\lambda ^i_j&{}\sum ^{N-1}_{i=0}A_{23}\lambda ^i_j&{}\sum ^{N-1}_{i=0}A_{24}\lambda ^i_j&{}\sum ^{N-1}_{i=0}A_{25}\lambda ^i_j\\ \sum ^{N-1}_{i=0}A_{31}\lambda ^i_j&{}\sum ^{N-1}_{i=0}A_{32}\lambda ^i_j&{}\lambda ^{N}+\sum ^{N-1}_{i=0}A_{33}\lambda ^i_j&{}\sum ^{N-1}_{i=0}A_{34}\lambda ^i_j&{}\sum ^{N-1}_{i=0}A_{35}\lambda ^i_j\\ \sum ^{N-1}_{i=0}A_{41}\lambda ^i_j&{}\sum ^{N-1}_{i=0}A_{42}\lambda ^i_j&{}\sum ^{N-1}_{i=0}A_{43}\lambda ^i_j&{}\lambda ^{N}+\sum ^{N-1}_{i=0}A_{44\lambda ^i_j}&{}\sum ^{N-1}_{i=0}A_{45}\lambda ^i_j\\ \sum ^{N-1}_{i=0}A_{51}\lambda ^i_j&{}\sum ^{N-1}_{i=0}A_{52}\lambda ^i_j&{}\sum ^{N-1}_{i=0}A_{53}\lambda ^i_j&{}\sum ^{N-1}_{i=0}A_{54}\lambda ^i_j&{}\lambda ^{N}+\sum ^{N-1}_{i=0}A_{55}\lambda ^i_j \end{array}\right) , \end{array} \end{aligned}$$

and

30 $$\begin{aligned} \begin{array}{l} \left\{ \begin{array}{lllllllll} \sum _{i=0}^{N-1} \left(A_{11}^{(i)}+A_{12}^{(i)}M_j^{(1)}+A^{(i)}_{13}M^{(2)}_{j}+A^{(i)}_{14}M^{(3)}_{j}+A^{(i)}_{15}M^{(4)}_{j} \right)\lambda _j^{i}=-\lambda _j^{N},\\ \sum _{i=0}^{N-1} \left(A_{21}^{(i)}+A_{22}^{(i)}M_j^{(1)}+A^{(i)}_{23}M^{(2)}_{j}+A^{(i)}_{24}M^{(3)}_{j}+A^{(i)}_{25}M^{(4)}_{j} \right)\lambda _j^{i}=-M^{(1)}_j\lambda _j^{N},\\ \sum _{i=0}^{N-1} \left(A_{31}^{(i)}+A_{32}^{(i)}M_j^{(1)}+A^{(i)}_{33}M^{(2)}_{j}+A^{(i)}_{34}M^{(3)}_{j}+A^{(i)}_{35}M^{(4)}_{j} \right)\lambda _j^{i}=-M^{(2)}_j\lambda _j^{N},\\ \sum _{i=0}^{N-1}(A_{41}^{(i)}+A_{42}^{(i)}M_j^{(1)}+A^{(i)}_{43}M^{(2)}_{j}+A^{(i)}_{44}M^{(3)}_{j}+A^{(i)}_{45}M^{(4)}_{j})\lambda _j^{i}=-M^{(3)}_j\lambda _j^{N},\\ \sum _{i=0}^{N-1} \left(A_{51}^{(i)}+A_{52}^{(i)}M_j^{(1)}+A^{(i)}_{53}M^{(2)}_{j}+A^{(i)}_{54}M^{(3)}_{j}+A^{(i)}_{55}M^{(4)}_{j}\right)\lambda _j^{i}=-M^{(4)}_j\lambda _j^{N}. \end{array}\right. \end{array} \end{aligned}$$

The N-soliton solutions of Eq. (1) on zero wave backgrounds are obtained according to Cramer’s rule in Eq. (30) as following forms:

31 $$\begin{aligned} \begin{array}{l} \left\{ \begin{array}{lllllllll} {\widetilde{q}}_1 =2A^{(N-1)}_{21},\\ {\widetilde{q}}_2=2A^{\left(N-1 \right)}_{31},\\ {\widetilde{q}}_3=2A^{\left(N-1 \right)}_{41},\\ {\widetilde{q}}_4=2A^{\left(N-1 \right)}_{51}, \end{array}\right. \end{array} \end{aligned}$$

where

32 $$\begin{aligned} \begin{array}{l} A^{(N-1)}_{21}=\frac{\Delta A^{(N-1)}_{21}}{\Delta },~~ A^{(N-1)}_{31}=\frac{\Delta A^{(N-1)}_{31}}{\Delta },~~ A^{(N-1)}_{41}=\frac{\Delta A^{(N-1)}_{41}}{\Delta },~~ A^{(N-1)}_{51}=\frac{\Delta A^{(N-1)}_{51}}{\Delta }, \end{array} \end{aligned}$$

with

33 $$\begin{aligned} \begin{array}{l} \Delta =\left| \begin{array}{lllllllllllllllll} 1&{}e^{\theta _{1,1}}&{}e^{\theta _{1,2}}&{}e^{\theta _{1,3}}&{}e^{\theta _{1,4}}&{}...&{}\lambda ^{(N-1)}_1&{}\lambda ^{(N-1)}_1e^{\theta _{1,1}}&{}...&{}\lambda ^{(N-1)}_1e^{\theta _{1,4}}&{}\\ 1&{}e^{\theta _{2,1}}&{}e^{\theta _{2,2}}&{}e^{\theta _{2,3}}&{}e^{\theta _{2,4}}&{}...&{}\lambda ^{(N-1)}_2&{}\lambda ^{(N-1)}_2e^{\theta _{2,1}}&{}...&{}\lambda ^{(N-1)}_2e^{\theta _{2,4}}&{}\\ ...&{}...&{}...&{}...&{}...&{}...&{}...&{}...&{}...&{}...\\ 1&{}e^{\theta _{5N,1}}&{}e^{\theta _{5N,2}}&{}e^{\theta _{5N,3}}&{}e^{\theta _{5N,4}}&{}...&{}\lambda ^{(N-1)}_{5N}&{}\lambda ^{(N-1)}_{5N}e^{\theta _{5N,1}}&{}...&{}\lambda ^{(N-1)}_{5N}e^{\theta _{5N,4}} \end{array}\right| ,\\ \Delta A^{(N-1)}_{21}=\left| \begin{array}{llllllllllllllllllc} 1&{}e^{\theta _{1,1}}&{}e^{\theta _{1,2}}&{}e^{\theta _{1,3}}&{}e^{\theta _{1,4}}&{}...&{}-\lambda ^{(N)}_1e^{\theta _{1,1}}&{}\lambda ^{(N-1)}_1e^{\theta _{1,1}}&{}...&{}\lambda ^{(N-1)}_1e^{\theta _{1,4}}&{}\\ 1&{}e^{\theta _{2,1}}&{}e^{\theta _{2,2}}&{}e^{\theta _{2,3}}&{}e^{\theta _{2,4}}&{}...&{}-\lambda ^{(N)}_2e^{\theta _{2,1}}&{}\lambda ^{(N-1)}_2e^{\theta _{2,1}}&{}...&{}\lambda ^{(N-1)}_2e^{\theta _{2,4}}&{}\\ ...&{}...&{}...&{}...&{}...&{}...&{}...&{}...&{}...&{}...\\ 1&{}e^{\theta _{5N,1}}&{}e^{\theta _{5N,2}}&{}e^{\theta _{5N,3}}&{}e^{\theta _{5N,4}}&{}...&{}-\lambda ^{(N)}_{5N}e^{\theta _{5N,1}}&{}\lambda ^{(N-1)}_{5N}e^{\theta _{5N,1}}&{}...&{}\lambda ^{(N-1)}_{5N}e^{\theta _{5N,4}} \end{array}\right| ,\\ \Delta A^{(N-1)}_{31}=\left| \begin{array}{lllllllllllllllllllllc} 1&{}e^{\theta _{1,1}}&{}e^{\theta _{1,2}}&{}e^{\theta _{1,3}}&{}e^{\theta _{1,4}}&{}...&{}-\lambda ^{(N)}_1e^{\theta _{1,2}}&{}\lambda ^{(N-1)}_1e^{\theta _{1,1}}&{}...&{}\lambda ^{(N-1)}_1e^{\theta _{1,4}}&{}\\ 1&{}e^{\theta _{2,1}}&{}e^{\theta _{2,2}}&{}e^{\theta _{2,3}}&{}e^{\theta _{2,4}}&{}...&{}-\lambda ^{(N)}_2e^{\theta _{2,2}}&{}\lambda ^{(N-1)}_2e^{\theta _{2,1}}&{}...&{}\lambda ^{(N-1)}_2e^{\theta _{2,4}}&{}\\ ...&{}...&{}...&{}...&{}...&{}...&{}...&{}...&{}...&{}...\\ 1&{}e^{\theta _{5N,1}}&{}e^{\theta _{5N,2}}&{}e^{\theta _{5N,3}}&{}e^{\theta _{5N,4}}&{}...&{}-\lambda ^{(N)}_{5N}e^{\theta _{5N,2}}&{}\lambda ^{(N-1)}_{5N}e^{\theta _{5N,1}}&{}...&{}\lambda ^{(N-1)}_{5N}e^{\theta _{5N,4}} \end{array}\right| ,\\ \Delta A^{(N-1)}_{41}=\left| \begin{array}{lllllllllllllllllllllc} 1&{}e^{\theta _{1,1}}&{}e^{\theta _{1,2}}&{}e^{\theta _{1,3}}&{}e^{\theta _{1,4}}&{}...&{}-\lambda ^{(N)}_1e^{\theta _{1,3}}&{}\lambda ^{(N-1)}_1e^{\theta _{1,1}}&{}...&{}\lambda ^{(N-1)}_1e^{\theta _{1,4}}&{}\\ 1&{}e^{\theta _{2,1}}&{}e^{\theta _{2,2}}&{}e^{\theta _{2,3}}&{}e^{\theta _{2,4}}&{}...&{}-\lambda ^{(N)}_2e^{\theta _{2,3}}&{}\lambda ^{(N-1)}_2e^{\theta _{2,1}}&{}...&{}\lambda ^{(N-1)}_2e^{\theta _{2,4}}&{}\\ ...&{}...&{}...&{}...&{}...&{}...&{}...&{}...&{}...&{}...\\ 1&{}e^{\theta _{5N,1}}&{}e^{\theta _{5N,2}}&{}e^{\theta _{5N,3}}&{}e^{\theta _{5N,4}}&{}...&{}-\lambda ^{(N)}_{5N}e^{\theta _{5N,3}}&{}\lambda ^{(N-1)}_{5N}e^{\theta _{5N,1}}&{}...&{}\lambda ^{(N-1)}_{5N}e^{\theta _{5N,4}} \end{array}\right| ,\\ \Delta A^{(N-1)}_{51}=\left| \begin{array}{llllllllllllllllllc} 1&{}e^{\theta _{1,1}}&{}e^{\theta _{1,2}}&{}e^{\theta _{1,3}}&{}e^{\theta _{1,4}}&{}...&{}-\lambda ^{(N)}_1e^{\theta _{1,4}}&{}\lambda ^{(N-1)}_1e^{\theta _{1,1}}&{}...&{}\lambda ^{(N-1)}_1e^{\theta _{1,4}}&{}\\ 1&{}e^{\theta _{2,1}}&{}e^{\theta _{2,2}}&{}e^{\theta _{2,3}}&{}e^{\theta _{2,4}}&{}...&{}-\lambda ^{(N)}_2e^{\theta _{2,4}}&{}\lambda ^{(N-1)}_2e^{\theta _{2,1}}&{}...&{}\lambda ^{(N-1)}_2e^{\theta _{2,4}}&{}\\ ...&{}...&{}...&{}...&{}...&{}...&{}...&{}...&{}...&{}...\\ 1&{}e^{\theta _{5N,1}}&{}e^{\theta _{5N,2}}&{}e^{\theta _{5N,3}}&{}e^{\theta _{5N,4}}&{}...&{}-\lambda ^{(N)}_{5N}e^{\theta _{5N,4}}&{}\lambda ^{(N-1)}_{5N}e^{\theta _{5N,1}}&{}...&{}\lambda ^{(N-1)}_{5N}e^{\theta _{5N,4}} \end{array}\right| , \end{array} \end{aligned}$$

and $$ \theta _{1,1}=-2(i\lambda _1x+i\lambda ^2_1t+F_{1,1}),~ \theta _{1,2}=-2(i\lambda _1x+i\lambda ^2_1t+F_{2,1}),~ \theta _{1,3}=-2(i\lambda _1x+i\lambda ^2_1t+F_{3,1}), \theta _{1,4}=-2(i\lambda _1x+i\lambda ^2_1t+F_{4,1}),~\theta _{2,1}=-2(i\lambda _2x+i\lambda ^2_2t+F_{1,2}),~\theta _{2,2}=-2(i\lambda _2x+i\lambda ^2_2t+F_{2,2}), \theta _{2,3}=-2(i\lambda _2x+i\lambda ^2_2t+F_{3,2}),~\theta _{2,4}=-2(i\lambda _2x+i\lambda ^2_2t+F_{4,2}),~\theta _{5N,1}=-2(i\lambda _{5N}x+i\lambda ^2_{5N}t+F_{1,5N}), \theta _{5N,2}=-2(i\lambda _{5N}x+i\lambda ^2_{5N}t+F_{2,5N}),~\theta _{5N,3}=-2(i\lambda _{5N}x+i\lambda ^2_{5N}t+F_{3,5N}),~\theta _{5N,4}=-2(i\lambda _{5N}x+i\lambda ^2_{5N}t+F_{4,5N}).$$

In order to obtain 1-soliton solution of the four-component CNLS equations on zero wave backgrounds, we consider $N=1$ in Eqs. (29) and (30), and obtain the matrix *T*

34 $$\begin{aligned} \begin{array}{l} T=\left( \begin{array}{llllllll} \lambda +A^{(0)}_{11}&{}A^{(0)}_{12}&{}A^{(0)}_{13}&{}A^{(0)}_{14}&{}A^{(0)}_{15}\\ A^{(0)}_{21}&{}\lambda +A^{(0)}_{22}&{}A^{(0)}_{23}&{}A^{(0)}_{24}&{}A^{(0)}_{25}\\ A^{(0)}_{31}&{}A^{(0)}_{32}&{}\lambda +A^{(0)}_{33}&{}A^{(0)}_{34}&{}A^{(0)}_{35}\\ A^{(0)}_{41}&{}A^{(0)}_{42}&{}A^{(0)}_{43}&{}\lambda +A^{(0)}_{44}&{}A^{(0)}_{45}\\ A^{(0)}_{51}&{}A^{(0)}_{52}&{}A^{(0)}_{53}&{}A^{(0)}_{54}&{}\lambda +A^{(0)}_{55}\end{array}\right) , \end{array} \end{aligned}$$

and

35 $$\begin{aligned} \begin{array}{l} \left\{ \begin{array}{lllllllll} A^{(0)}_{11}+A^{(0)}_{12}M_{j}^{(1)}+A^{(0)}_{13}M_{j}^{(2)}+A^{(0)}_{14}M_{j}^{(3)}+A^{(0)}_{15}M_{j}^{(4)}=-\lambda _j,\\ A^{(0)}_{21}+A^{(0)}_{22}M_{j}^{(1)}+A^{(0)}_{23}M_{j}^{(2)}+A^{(0)}_{24}M_{j}^{(3)}+A^{(0)}_{25}M_{j}^{(4)}=-M^{(1)}_j\lambda _j,\\ A^{(0)}_{31}+A^{(0)}_{32}M_{j}^{(1)}+A^{(0)}_{33}M_{j}^{(2)}+A^{(0)}_{34}M_{j}^{(3)}+A^{(0)}_{35}M_{j}^{(4)}=-M^{(2)}_j\lambda _j,\\ A^{(0)}_{41}+A^{(0)}_{42}M_{j}^{(1)}+A^{(0)}_{43}M_{j}^{(2)}+A^{(0)}_{44}M_{j}^{(3)}+A^{(0)}_{45}M_{j}^{(4)}=-M^{(3)}_j\lambda _j,\\ A^{(0)}_{51}+A^{(0)}_{52}M_{j}^{(1)}+A^{(0)}_{53}M_{j}^{(2)}+A^{(0)}_{54}M_{j}^{(3)}+A^{(0)}_{55}M_{j}^{(4)}=-M^{(4)}_j\lambda _j. \end{array}\right. \end{array} \end{aligned}$$

According to Eq. (35) and Cramer’s rule, we can derive the systems

36 $$\begin{aligned} \begin{array}{l} \Delta _1=\left| \begin{array}{lllllllll} 1&{}e^{-2\rho _{1,1}}&{}e^{-2\rho _{2,1}}&{}e^{-2\rho _{3,1}}&{}e^{-2\rho _{4,1}}\\ 1&{}e^{-2\rho _{1,2}}&{}e^{-2\rho _{2,2}}&{}e^{-2\rho _{3,2}}&{}e^{-2\rho _{4,2}}\\ 1&{}e^{-2\rho _{1,3}}&{}e^{-2\rho _{2,3}}&{}e^{-2\rho _{3,3}}&{}e^{-2\rho _{4,3}}\\ 1&{}e^{-2\rho _{1,4}}&{}e^{-2\rho _{2,4}}&{}e^{-2\rho _{3,4}}&{}e^{-2\rho _{4,4}}\\ 1&{}e^{-2\rho _{1,5}}&{}e^{-2\rho _{2,5}}&{}e^{-2\rho _{3,5}}&{}e^{-2\rho _{4,5}} \end{array}\right| , \\ \Delta A^{(0)}_{21}=\left| \begin{array}{lllllllll} -\lambda _1e^{-2\rho _{1,1}}&{}e^{-2\rho _{1,1}}&{}e^{-2\rho _{2,1}}&{}e^{-2\rho _{3,1}}&{}e^{-2\rho _{4,1}}\\ -\lambda _2e^{-2\rho _{1,2}}&{}e^{-2\rho _{1,2}}&{}e^{-2\rho _{2,2}}&{}e^{-2\rho _{3,2}}&{}e^{-2\rho _{4,2}}\\ -\lambda _3e^{-2\rho _{1,3}}&{}e^{-2\rho _{1,3}}&{}e^{-2\rho _{2,3}}&{}e^{-2\rho _{3,3}}&{}e^{-2\rho _{4,3}}\\ -\lambda _4e^{-2\rho _{1,4}}&{}e^{-2\rho _{1,4}}&{}e^{-2\rho _{2,4}}&{}e^{-2\rho _{3,4}}&{}e^{-2\rho _{4,4}}\\ -\lambda _5e^{-2\rho _{1,5}}&{}e^{-2\rho _{1,5}}&{}e^{-2\rho _{2,5}}&{}e^{-2\rho _{3,5}}&{}e^{-2\rho _{4,5}} \end{array}\right| , \\ \Delta A^{(0)}_{31}=\left| \begin{array}{lllllllllll} -\lambda _1e^{-2\rho _{2,1}}&{}e^{-2\rho _{1,1}}&{}e^{-2\rho _{2,1}}&{}e^{-2\rho _{3,1}}&{}e^{-2\rho _{4,1}}\\ -\lambda _2e^{-2\rho _{2,2}}&{}e^{-2\rho _{1,2}}&{}e^{-2\rho _{2,2}}&{}e^{-2\rho _{3,2}}&{}e^{-2\rho _{4,2}}\\ -\lambda _3e^{-2\rho _{2,3}}&{}e^{-2\rho _{1,3}}&{}e^{-2\rho _{2,3}}&{}e^{-2\rho _{3,3}}&{}e^{-2\rho _{4,3}}\\ -\lambda _4e^{-2\rho _{2,4}}&{}e^{-2\rho _{1,4}}&{}e^{-2\rho _{2,4}}&{}e^{-2\rho _{3,4}}&{}e^{-2\rho _{4,4}}\\ -\lambda _5e^{-2\rho _{2,5}}&{}e^{-2\rho _{1,5}}&{}e^{-2\rho _{2,5}}&{}e^{-2\rho _{3,5}}&{}e^{-2\rho _{4,5}} \end{array}\right| , \\ \Delta A^{(0)}_{41}= \left| \begin{array}{lllllllllllll} -\lambda _1e^{-2\rho _{3,1}}&{}e^{-2\rho _{1,1}}&{}e^{-2\rho _{2,1}}&{}e^{-2\rho _{3,1}}&{}e^{-2\rho _{4,1}}\\ -\lambda _2e^{-2\rho _{3,2}}&{}e^{-2\rho _{1,2}}&{}e^{-2\rho _{2,2}}&{}e^{-2\rho _{3,2}}&{}e^{-2\rho _{4,2}}\\ -\lambda _3e^{-2\rho _{3,3}}&{}e^{-2\rho _{1,3}}&{}e^{-2\rho _{2,3}}&{}e^{-2\rho _{3,3}}&{}e^{-2\rho _{4,3}}\\ -\lambda _4e^{-2\rho _{3,4}}&{}e^{-2\rho _{1,4}}&{}e^{-2\rho _{2,4}}&{}e^{-2\rho _{3,4}}&{}e^{-2\rho _{4,4}}\\ -\lambda _5e^{-2\rho _{3,5}}&{}e^{-2\rho _{1,5}}&{}e^{-2\rho _{2,5}}&{}e^{-2\rho _{3,5}}&{}e^{-2\rho _{4,5}} \end{array}\right| ,\\ \Delta A^{(0)}_{51}=\left| \begin{array}{llllllllllll} -\lambda _1e^{-2\rho _{4,1}}&{}e^{-2\rho _{1,1}}&{}e^{-2\rho _{2,1}}&{}e^{-2\rho _{3,1}}&{}e^{-2\rho _{4,1}}\\ -\lambda _2e^{-2\rho _{4,2}}&{}e^{-2\rho _{1,2}}&{}e^{-2\rho _{2,2}}&{}e^{-2\rho _{3,2}}&{}e^{-2\rho _{4,2}}\\ -\lambda _3e^{-2\rho _{4,3}}&{}e^{-2\rho _{1,3}}&{}e^{-2\rho _{2,3}}&{}e^{-2\rho _{3,3}}&{}e^{-2\rho _{4,3}}\\ -\lambda _4e^{-2\rho _{4,4}}&{}e^{-2\rho _{1,4}}&{}e^{-2\rho _{2,4}}&{}e^{-2\rho _{3,4}}&{}e^{-2\rho _{4,4}}\\ -\lambda _5e^{-2\rho _{4,5}}&{}e^{-2\rho _{1,5}}&{}e^{-2\rho _{2,5}}&{}e^{-2\rho _{3,5}}&{}e^{-2\rho _{4,5}} \end{array}\right| .\\ \end{array} \end{aligned}$$

here $$\rho _{i,1}=i\lambda _1x+i\lambda _1^2t+F_{i,1}$$, $$\rho _{i,2}=i\lambda _2x+i\lambda _2^2t+F_{i,2}$$, $$\rho _{i,3}=i\lambda _3x+i\lambda _3^2t+F_{i,3}$$, $$\rho _{i,4}=i\lambda _4x+i\lambda _4^2t+F_{i,4}$$, $$\rho _{i,1}=i\lambda _5x+i\lambda _5^2t+F_{i,5}$$, $$i=1,2,3,4$$.

Based on the Eq. (32), we can obtain the following systems

37 $$\begin{aligned} \begin{array}{l} A^{(0)}_{21}=\frac{\Delta A^{(0)}_{21}}{\Delta _1}, A^{(0)}_{31}=\frac{\Delta A^{(0)}_{31}}{\Delta _1}, A^{(0)}_{41}=\frac{\Delta A^{(0)}_{41}}{\Delta _1}, A^{(0)}_{51}=\frac{\Delta A^{(0)}_{51}}{\Delta _1}, \end{array} \end{aligned}$$

the analytic 1-soliton solutions of the four-component CNLS equations are obtained on zero wave backgrounds by Darboux transformation as following

38 $$\begin{aligned} \begin{array}{l} \left\{ \begin{array}{lllllllll} {\widetilde{q}}_1=2A^{(0)}_{21},\\ {\widetilde{q}}_2=2A^{(0)}_{31},\\ {\widetilde{q}}_3=2A^{(0)}_{41},\\ {\widetilde{q}}_4=2A^{(0)}_{51}. \end{array}\right. \end{array} \end{aligned}$$

In order to obtain the 2-soliton solutions of the four-component coupled NLS equations (1) on zero wave backgrounds, we consider $N=2$ in Eqs. (29) and (30), and obtain the matrix *T*

39 $$\begin{aligned} \begin{array}{l} T=\left( \begin{array}{lllllllllllllllll} \lambda ^2+A^{(1)}_{11}&{}A^{(1)}_{12}&{}A^{(1)}_{13}&{}A^{(1)}_{14}&{}A^{(1)}_{15}\\ A^{(1)}_{21}&{}\lambda ^2+A^{(1)}_{22}&{}A^{(1)}_{23}&{}A^{(1)}_{24}&{}A^{(1)}_{25}\\ A^{(1)}_{31}&{}A^{(1)}_{32}&{}\lambda ^2+A^{(1)}_{33}&{}A^{(1)}_{34}&{}A^{(1)}_{35}\\ A^{(1)}_{41}&{}A^{(1)}_{42}&{}A^{(1)}_{43}&{}\lambda ^2+A^{(1)}_{44}&{}A^{(1)}_{45}\\ A^{(1)}_{51}&{}A^{(1)}_{52}&{}A^{(1)}_{53}&{}A^{(0)}_{54}&{}\lambda ^2+A^{(1)}_{55}\end{array}\right) , \end{array} \end{aligned}$$

and

40 $$\begin{aligned} \begin{array}{l} \left\{ \begin{array}{lllllllll} A^{(1)}_{11}+A^{(1)}_{12}M_{j}^{(1)}+A^{(1)}_{13}M_{j}^{(2)}+A^{(1)}_{14}M_{j}^{(3)}+A^{(1)}_{15}M_{j}^{(4)}=-\lambda ^2_j,\\ A^{(1)}_{21}+A^{(1)}_{22}M_{j}^{(1)}+A^{(1)}_{23}M_{j}^{(2)}+A^{(1)}_{24}M_{j}^{(3)}+A^{(1)}_{25}M_{j}^{(4)}=-M^{(1)}_j\lambda ^2_j,\\ A^{(1)}_{31}+A^{(1)}_{32}M_{j}^{(1)}+A^{(1)}_{33}M_{j}^{(2)}+A^{(1)}_{34}M_{j}^{(3)}+A^{(1)}_{35}M_{j}^{(4)}=-M^{(2)}_j\lambda ^2_j,\\ A^{(1)}_{41}+A^{(1)}_{42}M_{j}^{(1)}+A^{(1)}_{43}M_{j}^{(2)}+A^{(1)}_{44}M_{j}^{(3)}+A^{(1)}_{45}M_{j}^{(4)}=-M^{(3)}_j\lambda ^2_j,\\ A^{(1)}_{51}+A^{(1)}_{52}M_{j}^{(1)}+A^{(1)}_{53}M_{j}^{(2)}+A^{(1)}_{54}M_{j}^{(3)}+A^{(1)}_{55}M_{j}^{(4)}=-M^{(4)}_j\lambda ^2_j. \end{array}\right. \end{array} \end{aligned}$$

According to Eq. (40) and Cramer’s rule, we can derive the systems

$$\begin{aligned} \Delta _2=\left| \begin{array}{llllllllllll} 1&{}e^{\theta _{1,1}}&{}e^{\theta _{1,2}}&{}e^{\theta _{1,3}}&{}e^{\theta _{1,4}}&{}\lambda _1&{}\lambda _1e^{\theta _{1,1}}&{}\lambda _1e^{\theta _{1,2}}&{}\lambda _1e^{\theta _{1,3}}&{}\lambda _1e^{\theta _{1,4}}\\ 1&{}e^{\theta _{2,1}}&{}e^{\theta _{2,2}}&{}e^{\theta _{2,3}}&{}e^{\theta _{2,4}}&{}\lambda _2&{}\lambda _2e^{\theta _{2,1}}&{}\lambda _2e^{\theta _{2,2}}&{}\lambda _2e^{\theta _{2,3}}&{}\lambda _2e^{\theta _{2,4}}\\ ...&{}...&{}...&{}...&{}...&{}...&{}...&{}...&{}...&{}...\\ 1&{}e^{\theta _{10,1}}&{}e^{\theta _{10,2}}&{}e^{\theta _{10,3}}&{}e^{\theta _{10,4}}&{}\lambda _{10}&{}\lambda _{10}e^{\theta _{10,1}}&{}\lambda _{10}e^{\theta _{10,2}}&{}\lambda _{10}e^{\theta _{10,3}}&{}\lambda _{10}e^{\theta _{10,4}} \end{array}\right| , \\ \Delta A^{(1)}_{21}=\left| \begin{array}{llllllllllll} 1&{}e^{\theta _{1,1}}&{}e^{\theta _{1,2}}&{}e^{\theta _{1,3}}&{}e^{\theta _{1,4}}&{}-\lambda _1^2e^{\theta _{1,1}}&{}\lambda _1e^{\theta _{1,1}}&{}\lambda _1e^{\theta _{1,2}}&{}\lambda _1e^{\theta _{1,3}}&{}\lambda _1e^{\theta _{1,4}}\\ 1&{}e^{\theta _{2,1}}&{}e^{\theta _{2,2}}&{}e^{\theta _{2,3}}&{}e^{\theta _{2,4}}&{}-\lambda _2^2e^{\theta _{2,1}}&{}\lambda _2e^{\theta _{2,1}}&{}\lambda _2e^{\theta _{2,2}}&{}\lambda _2e^{\theta _{2,3}}&{}\lambda _2e^{\theta _{2,4}}\\ ...&{}...&{}...&{}...&{}...&{}...&{}...&{}...&{}...&{}...\\ 1&{}e^{\theta _{10,1}}&{}e^{\theta _{10,2}}&{}e^{\theta _{10,3}}&{}e^{\theta _{10,4}}&{}-\lambda _{10}^2e^{\theta _{10,1}}&{}\lambda _{10}e^{\theta _{10,1}}&{}\lambda _{10}e^{\theta _{10,2}}&{}\lambda _{10}e^{\theta _{10,3}}&{}\lambda _{10}e^{\theta _{10,4}} \end{array}\right| , \\ \Delta A^{(1)}_{31}=\left| \begin{array}{llllllllllll} 1&{}e^{\theta _{1,1}}&{}e^{\theta _{1,2}}&{}e^{\theta _{1,3}}&{}e^{\theta _{1,4}}&{}-\lambda _1^2e^{\theta _{1,2}}&{}\lambda _1e^{\theta _{1,1}}&{}\lambda _1e^{\theta _{1,2}}&{}\lambda _1e^{\theta _{1,3}}&{}\lambda _1e^{\theta _{1,4}}\\ 1&{}e^{\theta _{2,1}}&{}e^{\theta _{2,2}}&{}e^{\theta _{2,3}}&{}e^{\theta _{2,4}}&{}-\lambda _2^2e^{\theta _{2,2}}&{}\lambda _2e^{\theta _{2,1}}&{}\lambda _2e^{\theta _{2,2}}&{}\lambda _2e^{\theta _{2,3}}&{}\lambda _2e^{\theta _{2,4}}\\ ...&{}...&{}...&{}...&{}...&{}...&{}...&{}...&{}...&{}...\\ 1&{}e^{\theta _{10,1}}&{}e^{\theta _{10,2}}&{}e^{\theta _{10,3}}&{}e^{\theta _{10,4}}&{}-\lambda _{10}^2e^{\theta _{10,2}}&{}\lambda _{10}e^{\theta _{10,1}}&{}\lambda _{10}e^{\theta _{10,2}}&{}\lambda _{10}e^{\theta _{10,3}}&{}\lambda _{10}e^{\theta _{10,4}} \end{array}\right| , \\ \Delta A^{(1)}_{41}=\left| \begin{array}{llllllllllll} 1&{}e^{\theta _{1,1}}&{}e^{\theta _{1,2}}&{}e^{\theta _{1,3}}&{}e^{\theta _{1,4}}&{}-\lambda _1^2e^{\theta _{1,3}}&{}\lambda _1e^{\theta _{1,1}}&{}\lambda _1e^{\theta _{1,2}}&{}\lambda _1e^{\theta _{1,3}}&{}\lambda _1e^{\theta _{1,4}}\\ 1&{}e^{\theta _{2,1}}&{}e^{\theta _{2,2}}&{}e^{\theta _{2,3}}&{}e^{\theta _{2,4}}&{}-\lambda _2^2e^{\theta _{2,3}}&{}\lambda _2e^{\theta _{2,1}}&{}\lambda _2e^{\theta _{2,2}}&{}\lambda _2e^{\theta _{2,3}}&{}\lambda _2e^{\theta _{2,4}}\\ ...&{}...&{}...&{}...&{}...&{}...&{}...&{}...&{}...&{}...\\ 1&{}e^{\theta _{10,1}}&{}e^{\theta _{10,2}}&{}e^{\theta _{10,3}}&{}e^{\theta _{10,4}}&{}-\lambda _{10}^2e^{\theta _{10,3}}&{}\lambda _{10}e^{\theta _{10,1}}&{}\lambda _{10}e^{\theta _{10,2}}&{}\lambda _{10}e^{\theta _{10,3}}&{}\lambda _{10}e^{\theta _{10,4}} \end{array}\right| , \end{aligned}$$

41 $$\begin{aligned} \begin{array}{l} \Delta A^{(1)}_{51}=\left| \begin{array}{llllllllllll} 1&{}e^{\theta _{1,1}}&{}e^{\theta _{1,2}}&{}e^{\theta _{1,3}}&{}e^{\theta _{1,4}}&{}-\lambda _1^2e^{\theta _{1,4}}&{}\lambda _1e^{\theta _{1,1}}&{}\lambda _1e^{\theta _{1,2}}&{}\lambda _1e^{\theta _{1,3}}&{}\lambda _1e^{\theta _{1,4}}\\ 1&{}e^{\theta _{2,1}}&{}e^{\theta _{2,2}}&{}e^{\theta _{2,3}}&{}e^{\theta _{2,4}}&{}-\lambda _2^2e^{\theta _{2,4}}&{}\lambda _2e^{\theta _{2,1}}&{}\lambda _2e^{\theta _{2,2}}&{}\lambda _2e^{\theta _{2,3}}&{}\lambda _2e^{\theta _{2,4}}\\ ...&{}...&{}...&{}...&{}...&{}...&{}...&{}...&{}...&{}...\\ 1&{}e^{\theta _{10,1}}&{}e^{\theta _{10,2}}&{}e^{\theta _{10,3}}&{}e^{\theta _{10,4}}&{}-\lambda _{10}^2e^{\theta _{10,4}}&{}\lambda _{10}e^{\theta _{10,1}}&{}\lambda _{10}e^{\theta _{10,2}}&{}\lambda _{10}e^{\theta _{10,3}}&{}\lambda _{10}e^{\theta _{10,4}} \end{array}\right| ,\\ \end{array} \end{aligned}$$

here $$\theta _{j,i}=-2(i\lambda _jx+i\lambda _j^2t+F_{(i,j)})$$,$$i=1,2,3,4$$,$$j=1,2,\ldots,10$$.

Based on Eq. (32), we can obtain the following systems

42 $$\begin{aligned} \begin{array}{l} A^{(1)}_{21}=\frac{\Delta A^{(1)}_{21}}{\Delta _2}, A^{(1)}_{31}=\frac{\Delta A^{(1)}_{31}}{\Delta _2}, A^{(1)}_{41}=\frac{\Delta A^{(1)}_{41}}{\Delta _2}, A^{(1)}_{51}=\frac{\Delta A^{(1)}_{51}}{\Delta _2}, \end{array} \end{aligned}$$

the analytic 2-soliton solutions of the four-component CNLS equation are obtained on zero wave backgrounds by Darboux transformation as following

43 $$\begin{aligned} \begin{array}{l} \left\{ \begin{array}{lllllllll} {\widetilde{q}}_1=2A^{(1)}_{21},\\ {\widetilde{q}}_2=2A^{(1)}_{31},\\ {\widetilde{q}}_3=2A^{(1)}_{41},\\ {\widetilde{q}}_4=2A^{(1)}_{51}. \end{array}\right. \end{array} \end{aligned}$$

#### N-soliton solutions on zero and plane wave backgrounds

   The *N*-soliton solution of the zero seed solution case is one of the simplest cases, however, the N-soliton solution formula of non-zero seed solution is more complicated with Darboux transformation. Because the non-zero solution must satisfy Eq. (1), we firstly give a set of seed solutions $q_1=e^{-it}, q_2=q_3=q_4=0$ and substitute these solutions into Eqs. (4) and (5), which can get the basic solutions for CNLS equations:

44 $$\begin{aligned} \begin{array}{l} \varphi =\left( \begin{array}{llllllll}He^{(\sqrt{1-\lambda ^2})x+it} \\ 0\\ 0\\ 0\\ 0\end{array}\right) , \phi =\left( \begin{array}{llllllll}0\\ K(\lambda +i\sqrt{1-\lambda ^2})e^{(\sqrt{1-\lambda ^2})x-it} \\ 0\\ 0\\ 0\end{array}\right) , \eta =\left( \begin{array}{llllllll}0\\ 0\\ e^{-i\lambda x-i\lambda ^2t} \\ 0\\ 0\end{array}\right) ,\\ \xi =\left( \begin{array}{llllllll}0\\ 0\\ 0\\ e^{-i\lambda x-i\lambda ^2t} \\ 0\end{array}\right) , \varepsilon =\left( \begin{array}{llllllll}0\\ 0\\ 0\\ 0\\ e^{-i\lambda x-i\lambda ^2t}\end{array}\right) . \end{array} \end{aligned}$$

with $$H=\sqrt{\frac{\lambda ^2+\frac{1}{2}}{\lambda ^2-\frac{1}{2}}}K$$.

Substituting Eq. (44) into Eq. (8), we give rise to

45 $$\begin{aligned} \begin{array}{l} M_j^{(1)}=\frac{K \left( \lambda +i\sqrt{1-\lambda ^2}\right) }{H}e^{-2it+G_{1,j}}, M_j^{(2)}=\frac{1}{H}e^{\left( -i\lambda -\sqrt{1-\lambda ^2}\right) x- \left( i\lambda ^2+i \right) t+G_{2,j})},\\ M_j^{(3)}=\frac{1}{H}e^{\left( -i\lambda -\sqrt{1-\lambda ^2}\right) x- \left( i\lambda ^2+i \right) t+G_{3,j})}, M_j^{(4)}=\frac{1}{H}e^{\left( -i\lambda -\sqrt{1-\lambda ^2}\right) x- \left( i\lambda ^2+i \right) t+G_{4,j})}, \end{array} \end{aligned}$$

where $$e^{G_{1,j}}=v_j^{(11)}, e^{G_{2,j}}=v_j^{(22)}, e^{G_{3,j}}=v_j^{(33)}, e^{G_{4,j}}=v_j^{(44)}$$, $$1\le j\le 5N$$.

In order to obtain the N-soliton solutions of Eq. (1) on zero and plane wave backgrounds, we consider matrix *T* as following:

46 $$\begin{aligned} \begin{array}{l} T=\left( \begin{array}{llllllll}\lambda ^{N}+\sum ^{N-1}_{i=0}A_{11}\lambda ^i_j&{}\sum ^{N-1}_{i=0}A_{12}\lambda ^i_j&{}\sum ^{N-1}_{i=0}A_{13}\lambda ^i_j&{}\sum ^{N-1}_{i=0}A_{14}\lambda ^i_j&{}\sum ^{N-1}_{i=0}A_{15}\lambda ^i_j\\ \sum ^{N-1}_{i=0}A_{21}\lambda ^i_j&{}\lambda ^{N}+\sum ^{N-1}_{i=0}A_{22}\lambda ^i_j&{}\sum ^{N-1}_{i=0}A_{23}\lambda ^i_j&{}\sum ^{N-1}_{i=0}A_{24}\lambda ^i_j&{}\sum ^{N-1}_{i=0}A_{25}\lambda ^i_j\\ \sum ^{N-1}_{i=0}A_{31}\lambda ^i_j&{}\sum ^{N-1}_{i=0}A_{32}\lambda ^i_j&{}\lambda ^{N}+\sum ^{N-1}_{i=0}A_{33}\lambda ^i_j&{}\sum ^{N-1}_{i=0}A_{34}\lambda ^i_j&{}\sum ^{N-1}_{i=0}A_{35}\lambda ^i_j\\ \sum ^{N-1}_{i=0}A_{41}\lambda ^i_j&{}\sum ^{N-1}_{i=0}A_{42}\lambda ^i_j&{}\sum ^{N-1}_{i=0}A_{43}\lambda ^i_j&{}\lambda ^{N}+\sum ^{N-1}_{i=0}A_{44\lambda ^i_j}&{}\sum ^{N-1}_{i=0}A_{45}\lambda ^i_j\\ \sum ^{N-1}_{i=0}A_{51}\lambda ^i_j&{}\sum ^{N-1}_{i=0}A_{52}\lambda ^i_j&{}\sum ^{N-1}_{i=0}A_{53}\lambda ^i_j&{}\sum ^{N-1}_{i=0}A_{54}\lambda ^i_j&{}\lambda ^{N}+\sum ^{N-1}_{i=0}A_{55}\lambda ^i_j \end{array}\right) , \end{array} \end{aligned}$$

and

47 $$\begin{aligned} \begin{array}{l} \left\{ \begin{array}{lllllllll} \sum _{i=0}^{N-1}(A_{11}^{(i)}+A_{12}^{(i)}M_j^{(1)}+A^{(i)}_{13}M^{(2)}_{j}+A^{(i)}_{14}M^{(3)}_{j}+A^{(i)}_{15}M^{(4)}_{j})\lambda _j^{i}=-\lambda _j^{N},\\ \sum _{i=0}^{N-1}(A_{21}^{(i)}+A_{22}^{(i)}M_j^{(1)}+A^{(i)}_{23}M^{(2)}_{j}+A^{(i)}_{24}M^{(3)}_{j}+A^{(i)}_{25}M^{(4)}_{j})\lambda _j^{i}=-M^{(1)}_j\lambda _j^{N},\\ \sum _{i=0}^{N-1}(A_{31}^{(i)}+A_{32}^{(i)}M_j^{(1)}+A^{(i)}_{33}M^{(2)}_{j}+A^{(i)}_{34}M^{(3)}_{j}+A^{(i)}_{35}M^{(4)}_{j})\lambda _j^{i}=-M^{(2)}_j\lambda _j^{N},\\ \sum _{i=0}^{N-1}(A_{41}^{(i)}+A_{42}^{(i)}M_j^{(1)}+A^{(i)}_{43}M^{(2)}_{j}+A^{(i)}_{44}M^{(3)}_{j}+A^{(i)}_{45}M^{(4)}_{j})\lambda _j^{i}=-M^{(3)}_j\lambda _j^{N},\\ \sum _{i=0}^{N-1}(A_{51}^{(i)}+A_{52}^{(i)}M_j^{(1)}+A^{(i)}_{53}M^{(2)}_{j}+A^{(i)}_{54}M^{(3)}_{j}+A^{(i)}_{55}M^{(4)}_{j})\lambda _j^{i}=-M^{(4)}_j\lambda _j^{N}. \end{array}\right. \end{array} \end{aligned}$$

The N-soliton solutions of Eq. (1) on zero and plane wave backgrounds are obtained according to Cramer’s rule in Eq. (47) as following forms:

48 $$\begin{aligned} \begin{array}{l} \left\{ \begin{array}{lllllllll} {\widetilde{q}}_1=e^{-it}+2A^{(N-1)}_{21},\\ {\widetilde{q}}_2=2A^{(N-1)}_{31},\\ {\widetilde{q}}_3=2A^{(N-1)}_{41},\\ {\widetilde{q}}_4=2A^{(N-1)}_{51}, \end{array}\right. \end{array} \end{aligned}$$

where

49 $$\begin{aligned} \begin{array}{l} A^{(N-1)}_{21}=\frac{\Delta A^{(N-1)}_{21}}{\Delta },~~ A^{(N-1)}_{31}=\frac{\Delta A^{(N-1)}_{31}}{\Delta },~~ A^{(N-1)}_{41}=\frac{\Delta A^{(N-1)}_{41}}{\Delta },~~ A^{(N-1)}_{51}=\frac{\Delta A^{(N-1)}_{51}}{\Delta }, \end{array} \end{aligned}$$

with

50 $$\begin{aligned} \begin{array}{l} \Delta =\left| \begin{array}{lllllllllllllllll} 1&{}\omega _1e^{\theta _{1,1}}&{}\frac{1}{H}e^{\theta _{1,2}}&{}\frac{1}{H}e^{\theta _{1,3}}&{}\frac{1}{H}e^{\theta _{1,4}}&{}...&{}\lambda ^{(N-1)}_1&{}\omega _1\lambda ^{(N-1)}_1e^{\theta _{1,1}}&{}...&{}\frac{1}{H}\lambda ^{(N-1)}_1e^{\theta _{1,4}}&{}\\ 1&{}\omega _2e^{\theta _{2,1}}&{}\frac{1}{H}e^{\theta _{2,2}}&{}\frac{1}{H}e^{\theta _{2,3}}&{}\frac{1}{H}e^{\theta _{2,4}}&{}...&{}\lambda ^{(N-1)}_2&{}\omega _2\lambda ^{(N-1)}_2e^{\theta _{2,1}}&{}...&{}\frac{1}{H}\lambda ^{(N-1)}_2e^{\theta _{2,4}}&{}\\ ...&{}...&{}...&{}...&{}...&{}...&{}...&{}...&{}...&{}...\\ 1&{}\omega _{5N}e^{\theta _{5N,1}}&{}\frac{1}{H}e^{\theta _{5N,2}}&{}\frac{1}{H}e^{\theta _{5N,3}}&{}\frac{1}{H}e^{\theta _{5N,4}}&{}...&{}\lambda ^{(N-1)}_{5N}&{}\omega _{5N}\lambda ^{(N-1)}_{5N}e^{\theta _{5N,1}}&{}...&{}\frac{1}{H}\lambda ^{(N-1)}_{5N}e^{\theta _{5N,4}} \end{array}\right| ,\\ \Delta A^{(N-1)}_{21}=\left| \begin{array}{lllllllllllllllllll} 1&{}\omega _1e^{\theta _{1,1}}&{}\frac{1}{H}e^{\theta _{1,2}}&{}...&{}-\omega _1\lambda ^{(N)}_1e^{\theta _{1,1}}&{}\omega _1\lambda ^{(N-1)}_1e^{\theta _{1,1}}&{}...&{}\frac{1}{H}\lambda ^{(N-1)}_1e^{\theta _{1,4}}&{}\\ 1&{}\omega _2e^{\theta _{2,1}}&{}\frac{1}{H}e^{\theta _{2,2}}&{}...&{}-\omega _2\lambda ^{(N)}_2e^{\theta _{2,1}}&{}\omega _2\lambda ^{(N-1)}_2e^{\theta _{2,1}}&{}...&{}\frac{1}{H}\lambda ^{(N-1)}_2e^{\theta _{2,4}}&{}\\ ...&{}...&{}...&{}...&{}...&{}...&{}...&{}...\\ 1&{}\omega _{5N}e^{\theta _{5N,1}}&{}\frac{1}{H}e^{\theta _{5N,2}}&{}...&{}-\omega _{5N}\lambda ^{(N)}_{5N}e^{\theta _{5N,1}}&{}-\omega _{5N}\lambda ^{(N-1)}_{5N}e^{\theta _{5N,1}}&{}...&{}\frac{1}{H}\lambda ^{(N-1)}_{5N}e^{\theta _{5N,4}} \end{array}\right| ,\\ \Delta A^{(N-1)}_{31}=\left| \begin{array}{llllllllllllllllllllll} 1&{}\omega _1e^{\theta _{1,1}}&{}\frac{1}{H}e^{\theta _{1,2}}&{}...&{}-\frac{1}{H}\lambda ^{(N)}_1e^{\theta _{1,2}}&{}\omega _1\lambda ^{(N-1)}_1e^{\theta _{1,1}}&{}...&{}\frac{1}{H}\lambda ^{(N-1)}_1e^{\theta _{1,4}}&{}\\ 1&{}\omega _2e^{\theta _{2,1}}&{}\frac{1}{H}e^{\theta _{2,2}}&{}...&{}-\frac{1}{H}\lambda ^{(N)}_2e^{\theta _{2,2}}&{}\omega _2\lambda ^{(N-1)}_2e^{\theta _{2,1}}&{}...&{}\frac{1}{H}\lambda ^{(N-1)}_2e^{\theta _{2,4}}&{}\\ ...&{}...&{}...&{}...&{}...&{}...&{}...&{}...\\ 1&{}\omega _{5N}e^{\theta _{5N,1}}&{}\frac{1}{H}e^{\theta _{5N,2}}&{}...&{}-\frac{1}{H}\lambda ^{(N)}_{5N}e^{\theta _{5N,2}}&{}\omega _{5N}\lambda ^{(N-1)}_{5N}e^{\theta _{5N,1}}&{}...&{}\frac{1}{H}\lambda ^{(N-1)}_{5N}e^{\theta _{5N,4}} \end{array}\right| ,\\ \Delta A^{(N-1)}_{41}=\left| \begin{array}{llllllllllllllllllllll} 1&{}\omega _1e^{\theta _{1,1}}&{}\frac{1}{H}e^{\theta _{1,2}}&{}...&{}-\frac{1}{H}\lambda ^{(N)}_1e^{\theta _{1,3}}&{}\omega _1\lambda ^{(N-1)}_1e^{\theta _{1,1}}&{}...&{}\frac{1}{H}\lambda ^{(N-1)}_1e^{\theta _{1,4}}&{}\\ 1&{}\omega _1e^{\theta _{2,1}}&{}\frac{1}{H}e^{\theta _{2,2}}&{}...&{}-\frac{1}{H}\lambda ^{(N)}_2e^{\theta _{2,3}}&{}\omega _2\lambda ^{(N-1)}_2e^{\theta _{2,1}}&{}...&{}\frac{1}{H}\lambda ^{(N-1)}_2e^{\theta _{2,4}}&{}\\ ...&{}...&{}...&{}...&{}...&{}...&{}...&{}...\\ 1&{}\omega _1e^{\theta _{5N,1}}&{}\frac{1}{H}e^{\theta _{5N,2}}&{}...&{}-\frac{1}{H}\lambda ^{(N)}_{5N}e^{\theta _{5N,3}}&{}\omega _{5N}\lambda ^{(N-1)}_{5N}e^{\theta _{5N,1}}&{}...&{}\frac{1}{H}\lambda ^{(N-1)}_{5N}e^{\theta _{5N,4}} \end{array}\right| ,\\ \Delta A^{(N-1)}_{51}=\left| \begin{array}{lllllllllllllllllll} 1&{}\omega _1e^{\theta _{1,1}}&{}\frac{1}{H}e^{\theta _{1,2}}&{}...&{}-\frac{1}{H}\lambda ^{(N)}_1e^{\theta _{1,4}}&{}\omega _1\lambda ^{(N-1)}_1e^{\theta _{1,1}}&{}...&{}\frac{1}{H}\lambda ^{(N-1)}_1e^{\theta _{1,4}}&{}\\ 1&{}\omega _2e^{\theta _{2,1}}&{}\frac{1}{H}e^{\theta _{2,2}}&{}...&{}-\frac{1}{H}\lambda ^{(N)}_2e^{\theta _{2,4}}&{}\omega _2\lambda ^{(N-1)}_2e^{\theta _{2,1}}&{}...&{}\frac{1}{H}\lambda ^{(N-1)}_2e^{\theta _{2,4}}&{}\\ ...&{}...&{}...&{}...&{}...&{}...&{}...&{}...\\ 1&{}\omega _{5N}e^{\theta _{5N,1}}&{}\frac{1}{H}e^{\theta _{5N,2}}&{}...&{}-\frac{1}{H}\lambda ^{(N)}_{5N}e^{\theta _{5N,4}}&{}\omega _{5N}\lambda ^{(N-1)}_{5N}e^{\theta _{5N,1}}&{}...&{}\frac{1}{H}\lambda ^{(N-1)}_{5N}e^{\theta _{5N,4}} \end{array}\right| , \end{array} \end{aligned}$$

with $\omega _j=\frac{K}{H}\left( \lambda _j+i\sqrt{1-\lambda ^2_j}\right)$, where *i* is an imaginary number, then $\theta _{j,1}=-2it+G_{(1,j)},\,\,\theta _{j,i}=\left(-i\lambda _j-\sqrt{1-\lambda ^2_j}\right)x-(i\lambda ^2_j+i)t+G_{(1,j)}$, where the ranges of the subscripts *j*, *i* of $\theta$ are $1\le i\le 4$, $1\le j\le 5N$.

In order to obtain 1-soliton solutions of the four-component coupled NLS equations on zero and plane wave backgrounds, we consider $N=1$ in Eqs. (46) and (47), and obtain the matrix *T*

51 $$\begin{aligned} \begin{array}{l} T=\left( \begin{array}{llllllll} \lambda +A^{(0)}_{11}&{}A^{(0)}_{12}&{}A^{(0)}_{13}&{}A^{(0)}_{14}&{}A^{(0)}_{15}\\ A^{(0)}_{21}&{}\lambda +A^{(0)}_{22}&{}A^{(0)}_{23}&{}A^{(0)}_{24}&{}A^{(0)}_{25}\\ A^{(0)}_{31}&{}A^{(0)}_{32}&{}\lambda +A^{(0)}_{33}&{}A^{(0)}_{34}&{}A^{(0)}_{35}\\ A^{(0)}_{41}&{}A^{(0)}_{42}&{}A^{(0)}_{43}&{}\lambda +A^{(0)}_{44}&{}A^{(0)}_{45}\\ A^{(0)}_{51}&{}A^{(0)}_{52}&{}A^{(0)}_{53}&{}A^{(0)}_{54}&{}\lambda +A^{(0)}_{55}\end{array}\right) , \end{array} \end{aligned}$$

and

52 $$\begin{aligned} \begin{array}{l} \left\{ \begin{array}{lllllllll} A^{(0)}_{11}+A^{(0)}_{12}M_{j}^{(1)}+A^{(0)}_{13}M_{j}^{(2)}+A^{(0)}_{14}M_{j}^{(3)}+A^{(0)}_{15}M_{j}^{(4)}=-\lambda _j,\\ A^{(0)}_{21}+A^{(0)}_{22}M_{j}^{(1)}+A^{(0)}_{23}M_{j}^{(2)}+A^{(0)}_{24}M_{j}^{(3)}+A^{(0)}_{25}M_{j}^{(4)}=-M^{(1)}_j\lambda _j,\\ A^{(0)}_{31}+A^{(0)}_{32}M_{j}^{(1)}+A^{(0)}_{33}M_{j}^{(2)}+A^{(0)}_{34}M_{j}^{(3)}+A^{(0)}_{35}M_{j}^{(4)}=-M^{(2)}_j\lambda _j,\\ A^{(0)}_{41}+A^{(0)}_{42}M_{j}^{(1)}+A^{(0)}_{43}M_{j}^{(2)}+A^{(0)}_{44}M_{j}^{(3)}+A^{(0)}_{45}M_{j}^{(4)}=-M^{(3)}_j\lambda _j,\\ A^{(0)}_{51}+A^{(0)}_{52}M_{j}^{(1)}+A^{(0)}_{53}M_{j}^{(2)}+A^{(0)}_{54}M_{j}^{(3)}+A^{(0)}_{55}M_{j}^{(4)}=-M^{(4)}_j\lambda _j. \end{array}\right. \end{array} \end{aligned}$$

According to Eq. (52) and Cramer’s rule, we can derive the systems

$$\begin{aligned} \Delta _1=\left| \begin{array}{llllllllllllll} 1&{}\omega _1e^{\theta _{1,1}}&{}\frac{1}{H}e^{\theta _{1,2}}&{}\frac{1}{H}e^{\theta _{1,3}}&{}\frac{1}{H}e^{\theta _{1,4}}\\ 1&{}\omega _2e^{\theta _{2,1}}&{}\frac{1}{H}e^{\theta _{2,2}}&{}\frac{1}{H}e^{\theta _{2,3}}&{}\frac{1}{H}e^{\theta _{2,4}}\\ 1&{}\omega _3e^{\theta _{3,1}}&{}\frac{1}{H}e^{\theta _{3,2}}&{}\frac{1}{H}e^{\theta _{3,3}}&{}\frac{1}{H}e^{\theta _{3,4}}\\ 1&{}\omega _4e^{\theta _{4,1}}&{}\frac{1}{H}e^{\theta _{4,2}}&{}\frac{1}{H}e^{\theta _{4,3}}&{}\frac{1}{H}e^{\theta _{4,4}}\\ 1&{}\omega _5e^{\theta _{5,1}}&{}\frac{1}{H}e^{\theta _{5,2}}&{}\frac{1}{H}e^{\theta _{5,3}}&{}\frac{1}{H}e^{\theta _{5,4}} \end{array}\right| ,~~\Delta A^{(0)}_{21}=\left| \begin{array}{lllllllllllllll} -\omega _1\lambda _1e^{\theta _{1,1}}&{}\omega _1e^{\theta _{1,1}}&{}\frac{1}{H}e^{\theta _{1,2}}&{}\frac{1}{H}e^{\theta _{1,3}}&{}\frac{1}{H}e^{\theta _{1,4}}\\ -\omega _2\lambda _2e^{\theta _{2,1}}&{}\omega _2e^{\theta _{2,1}}&{}\frac{1}{H}e^{\theta _{2,2}}&{}\frac{1}{H}e^{\theta _{2,3}}&{}\frac{1}{H}e^{\theta _{2,4}}\\ -\omega _3\lambda _3e^{\theta _{3,1}}&{}\omega _3e^{\theta _{3,1}}&{}\frac{1}{H}e^{\theta _{3,2}}&{}\frac{1}{H}e^{\theta _{3,3}}&{}\frac{1}{H}e^{\theta _{3,4}}\\ -\omega _4\lambda _4e^{\theta _{4,1}}&{}\omega _4e^{\theta _{4,1}}&{}\frac{1}{H}e^{\theta _{4,2}}&{}\frac{1}{H}e^{\theta _{4,3}}&{}\frac{1}{H}e^{\theta _{4,4}}\\ -\omega _5\lambda _5e^{\theta _{5,1}}&{}\omega _5e^{\theta _{5,1}}&{}\frac{1}{H}e^{\theta _{5,2}}&{}\frac{1}{H}e^{\theta _{5,3}}&{}\frac{1}{H}e^{\theta _{5,4}} \end{array}\right| , \\ \Delta A^{(0)}_{31}=\left| \begin{array}{llllllllllllllll} -\frac{1}{H}\lambda e^{\theta _{1,2}}&{}\omega _1e^{\theta _{1,1}}&{}\frac{1}{H}e^{\theta _{1,2}}&{}\frac{1}{H}e^{\theta _{1,3}}&{}\frac{1}{H}e^{\theta _{1,4}}\\ -\frac{1}{H}\lambda e^{\theta _{2,2}}&{}\omega _2e^{\theta _{2,1}}&{}\frac{1}{H}e^{\theta _{2,2}}&{}\frac{1}{H}e^{\theta _{2,3}}&{}\frac{1}{H}e^{\theta _{2,4}}\\ -\frac{1}{H}\lambda e^{\theta _{3,2}}&{}\omega _3e^{\theta _{3,1}}&{}\frac{1}{H}e^{\theta _{3,2}}&{}\frac{1}{H}e^{\theta _{3,3}}&{}\frac{1}{H}e^{\theta _{3,4}}\\ -\frac{1}{H}\lambda e^{\theta _{4,2}}&{}\omega _4e^{\theta _{4,1}}&{}\frac{1}{H}e^{\theta _{4,2}}&{}\frac{1}{H}e^{\theta _{4,3}}&{}\frac{1}{H}e^{\theta _{4,4}}\\ -\frac{1}{H}\lambda e^{\theta _{5,2}}&{}\omega _5e^{\theta _{5,1}}&{}\frac{1}{H}e^{\theta _{5,2}}&{}\frac{1}{H}e^{\theta _{5,3}}&{}\frac{1}{H}e^{\theta _{5,4}} \end{array}\right| , ~~~\Delta A^{(0)}_{41}= \left| \begin{array}{lllllllllllllllll} -\frac{1}{H}\lambda _1e^{\theta _{1,3}}&{}\omega _1e^{\theta _{1,1}}&{}\frac{1}{H}e^{\theta _{1,2}}&{}\frac{1}{H}e^{\theta _{1,3}}&{}\frac{1}{H}e^{\theta _{1,4}}\\ -\frac{1}{H}\lambda _2e^{\theta _{2,3}}&{}\omega _2e^{\theta _{2,1}}&{}\frac{1}{H}e^{\theta _{2,2}}&{}\frac{1}{H}e^{\theta _{2,3}}&{}\frac{1}{H}e^{\theta _{2,4}}\\ -\frac{1}{H}\lambda _3e^{\theta _{3,3}}&{}\omega _3e^{\theta _{3,1}}&{}\frac{1}{H}e^{\theta _{3,2}}&{}\frac{1}{H}e^{\theta _{3,3}}&{}\frac{1}{H}e^{\theta _{3,4}}\\ -\frac{1}{H}\lambda _4e^{\theta _{4,3}}&{}\omega _4e^{\theta _{4,1}}&{}\frac{1}{H}e^{\theta _{4,2}}&{}\frac{1}{H}e^{\theta _{4,3}}&{}\frac{1}{H}e^{\theta _{4,4}}\\ -\frac{1}{H}\lambda _5e^{\theta _{5,3}}&{}\omega _5e^{\theta _{5,1}}&{}\frac{1}{H}e^{\theta _{5,2}}&{}\frac{1}{H}e^{\theta _{5,3}}&{}\frac{1}{H}e^{\theta _{5,4}} \end{array}\right| , \end{aligned}$$

53 $$\begin{aligned} \begin{array}{l} \Delta A^{(0)}_{51}=\left| \begin{array}{lllllllllllllllll} -\frac{1}{H}\lambda _1e^{\theta _{1,4}}&{}\omega _1e^{\theta _{1,1}}&{}\frac{1}{H}e^{\theta _{1,2}}&{}\frac{1}{H}e^{\theta _{1,3}}&{}\frac{1}{H}e^{\theta _{1,4}}\\ -\frac{1}{H}\lambda _2e^{\theta _{2,4}}&{}\omega _2e^{\theta _{2,1}}&{}\frac{1}{H}e^{\theta _{2,2}}&{}\frac{1}{H}e^{\theta _{2,3}}&{}\frac{1}{H}e^{\theta _{2,4}}\\ -\frac{1}{H}\lambda _3e^{\theta _{3,4}}&{}\omega _3e^{\theta _{3,1}}&{}\frac{1}{H}e^{\theta _{3,2}}&{}\frac{1}{H}e^{\theta _{3,3}}&{}\frac{1}{H}e^{\theta _{3,4}}\\ -\frac{1}{H}\lambda _4e^{\theta _{4,4}}&{}\omega _4e^{\theta _{4,1}}&{}\frac{1}{H}e^{\theta _{4,2}}&{}\frac{1}{H}e^{\theta _{4,3}}&{}\frac{1}{H}e^{\theta _{4,4}}\\ -\frac{1}{H}\lambda _5e^{\theta _{5,4}}&{}\omega _5e^{\theta _{5,1}}&{}\frac{1}{H}e^{\theta _{5,2}}&{}\frac{1}{H}e^{\theta _{5,3}}&{}\frac{1}{H}e^{\theta _{5,4}} \end{array}\right| .\\ \end{array} \end{aligned}$$

here $\omega _j=\frac{K}{H}(\lambda _j+i\sqrt{1-\lambda ^2_j})$, the *i* is an imaginary number, then $\theta _{j,1}=-2it+G_{(1,j)}$, $\theta _{j,i}=(-i\lambda _j-\sqrt{1-\lambda ^2_j})x-(i\lambda ^2_j+i)t+G_{(1,j)}$, where the ranges of the subscripts *j* and *i* of $\theta$ are $1\le i\le 4$, $1\le j\le 5$.

Based on Eq. (49), we can obtain the following systems

54 $$\begin{aligned} \begin{array}{l} A^{(0)}_{21}=\frac{\Delta A^{(0)}_{21}}{\Delta _1}, A^{(0)}_{31}=\frac{\Delta A^{(0)}_{31}}{\Delta _1}, A^{(0)}_{41}=\frac{\Delta A^{(0)}_{41}}{\Delta _1}, A^{(0)}_{51}=\frac{\Delta A^{(0)}_{51}}{\Delta _1}, \end{array} \end{aligned}$$

the analytic 1-soliton solutions of the four-component CNLS equations are obtained on zero and plane wave backgrounds by Darboux transformation as following

55 $$\begin{aligned} \begin{array}{l} \left\{ \begin{array}{lllllllll} {\widetilde{q}}_1=e^{-it}+2A^{(0)}_{21},\\ {\widetilde{q}}_2=2A^{(0)}_{31},\\ {\widetilde{q}}_3=2A^{(0)}_{41},\\ {\widetilde{q}}_4=2A^{(0)}_{51}. \end{array}\right. \end{array} \end{aligned}$$

In order to obtain 2-soliton solutions of the four-component CNLS equations on zero and plane wave backgrounds, we consider $N=2$ in Eqs. (46) and (47), and obtain the matrix *T*

56 $$\begin{aligned} \begin{array}{l} T=\left( \begin{array}{llllllll} \lambda ^2+A^{(1)}_{11}&{}A^{(1)}_{12}&{}A^{(1)}_{13}&{}A^{(1)}_{14}&{}A^{(1)}_{15}\\ A^{(1)}_{21}&{}\lambda ^2+A^{(1)}_{22}&{}A^{(1)}_{23}&{}A^{(1)}_{24}&{}A^{(1)}_{25}\\ A^{(1)}_{31}&{}A^{(1)}_{32}&{}\lambda ^2+A^{(1)}_{33}&{}A^{(1)}_{34}&{}A^{(1)}_{35}\\ A^{(1)}_{41}&{}A^{(1)}_{42}&{}A^{(1)}_{43}&{}\lambda ^2+A^{(1)}_{44}&{}A^{(1)}_{45}\\ A^{(1)}_{51}&{}A^{(1)}_{52}&{}A^{(1)}_{53}&{}A^{(0)}_{54}&{}\lambda ^2+A^{(1)}_{55}\end{array}\right) , \end{array} \end{aligned}$$

and

57 $$\begin{aligned} \begin{array}{l} \left\{ \begin{array}{lllllllll} A^{(1)}_{11}+A^{(1)}_{12}M_{j}^{(1)}+A^{(1)}_{13}M_{j}^{(2)}+A^{(1)}_{14}M_{j}^{(3)}+A^{(1)}_{15}M_{j}^{(4)}=-\lambda ^2_j,\\ A^{(1)}_{21}+A^{(1)}_{22}M_{j}^{(1)}+A^{(1)}_{23}M_{j}^{(2)}+A^{(1)}_{24}M_{j}^{(3)}+A^{(1)}_{25}M_{j}^{(4)}=-M^{(1)}_j\lambda ^2_j,\\ A^{(1)}_{31}+A^{(1)}_{32}M_{j}^{(1)}+A^{(1)}_{33}M_{j}^{(2)}+A^{(1)}_{34}M_{j}^{(3)}+A^{(1)}_{35}M_{j}^{(4)}=-M^{(2)}_j\lambda ^2_j,\\ A^{(1)}_{41}+A^{(1)}_{42}M_{j}^{(1)}+A^{(1)}_{43}M_{j}^{(2)}+A^{(1)}_{44}M_{j}^{(3)}+A^{(1)}_{45}M_{j}^{(4)}=-M^{(3)}_j\lambda ^2_j,\\ A^{(1)}_{51}+A^{(1)}_{52}M_{j}^{(1)}+A^{(1)}_{53}M_{j}^{(2)}+A^{(1)}_{54}M_{j}^{(3)}+A^{(1)}_{55}M_{j}^{(4)}=-M^{(4)}_j\lambda ^2_j. \end{array}\right. \end{array} \end{aligned}$$

According to Eq. (57) and Cramer’s rule, we can derive the systems

$$\begin{aligned} \Delta _2=\left| \begin{array}{llllllllllll} 1&{}\omega _1e^{\theta _{1,1}}&{}\frac{1}{H}e^{\theta _{1,2}}&{}\frac{1}{H}e^{\theta _{1,3}}&{}\frac{1}{H}e^{\theta _{1,4}}&{}\lambda _1&{}\omega _1\lambda _1e^{\theta _{1,1}}&{}\frac{1}{H}\lambda _1e^{\theta _{1,2}}&{}\frac{1}{H}\lambda _1e^{\theta _{1,3}}&{}\frac{1}{H}\lambda _1e^{\theta _{1,4}}\\ 1&{}\omega _2e^{\theta _{2,1}}&{}\frac{1}{H}e^{\theta _{2,2}}&{}\frac{1}{H}e^{\theta _{2,3}}&{}\frac{1}{H}e^{\theta _{2,4}}&{}\lambda _2&{}\omega _2\lambda _2e^{\theta _{2,1}}&{}\frac{1}{H}\lambda _2e^{\theta _{2,2}}&{}\frac{1}{H}\lambda _2e^{\theta _{2,3}}&{}\frac{1}{H}\lambda _2e^{\theta _{2,4}}\\ ...&{}...&{}...&{}...&{}...&{}...&{}...&{}...&{}...&{}...\\ 1&{}\omega _{10}e^{\theta _{10,1}}&{}\frac{1}{H}e^{\theta _{10,2}}&{}\frac{1}{H}e^{\theta _{10,3}}&{}\frac{1}{H}e^{\theta _{10,4}}&{}\lambda _{10}&{}\omega _{10}\lambda _{10}e^{\theta _{10,1}}&{}\frac{1}{H}\lambda _{10}e^{\theta _{10,2}}&{}\frac{1}{H}\lambda _{10}e^{\theta _{10,3}}&{}\frac{1}{H}\lambda _{10}e^{\theta _{10,4}} \end{array}\right| , \\ \Delta A^{(1)}_{21}=\left| \begin{array}{llllllllllll} 1&{}\omega _1e^{\theta _{1,1}}&{}\frac{1}{H}e^{\theta _{1,2}}&{}\frac{1}{H}e^{\theta _{1,3}}&{}\frac{1}{H}e^{\theta _{1,4}}&{}-\omega -1\lambda _1^2e^{\theta _{1,1}}&{}\omega _1\lambda _1e^{\theta _{1,1}}&{}...&{}\frac{1}{H}\lambda _1e^{\theta _{1,4}}\\ 1&{}\omega _2e^{\theta _{2,1}}&{}\frac{1}{H}e^{\theta _{2,2}}&{}\frac{1}{H}e^{\theta _{2,3}}&{}\frac{1}{H}e^{\theta _{2,4}}&{}-\omega _2\lambda _2^2e^{\theta _{2,1}}&{}\omega _2\lambda _2e^{\theta _{2,1}}&{}...&{}\frac{1}{H}\lambda _2e^{\theta _{2,4}}\\ ...&{}...&{}...&{}...&{}...&{}...&{}...&{}...&{}...\\ 1&{}\omega _{10}e^{\theta _{10,1}}&{}\frac{1}{H}e^{\theta _{10,2}}&{}\frac{1}{H}e^{\theta _{10,3}}&{}\frac{1}{H}e^{\theta _{10,4}}&{}-\omega _{10}\lambda _{10}^2e^{\theta _{10,1}}&{}\omega _{10}\lambda _{10}e^{\theta _{10,1}}&{}...&{}\frac{1}{H}\lambda _{10}e^{\theta _{10,4}} \end{array}\right| , \end{aligned}$$

58 $$\begin{aligned} \begin{array}{l} \Delta A^{(1)}_{31}=\left| \begin{array}{llllllllllll} 1&{}\omega _1e^{\theta _{1,1}}&{}\frac{1}{H}e^{\theta _{1,2}}&{}\frac{1}{H}e^{\theta _{1,3}}&{}\frac{1}{H}e^{\theta _{1,4}}&{}-\frac{1}{H}\lambda _1^2e^{\theta _{1,2}}&{}\omega _1\lambda _1e^{\theta _{1,1}}&{}...&{}\frac{1}{H}\lambda _1e^{\theta _{1,4}}\\ 1&{}\omega _1e^{\theta _{2,1}}&{}\frac{1}{H}e^{\theta _{2,2}}&{}\frac{1}{H}e^{\theta _{2,3}}&{}\frac{1}{H}e^{\theta _{2,4}}&{}-\frac{1}{H}\lambda _2^2e^{\theta _{2,2}}&{}\omega _2\lambda _2e^{\theta _{2,1}}&{}...&{}\frac{1}{H}\lambda _2e^{\theta _{2,4}}\\ ...&{}...&{}...&{}...&{}...&{}...&{}...&{}...&{}...\\ 1&{}\omega _1e^{\theta _{10,1}}&{}\frac{1}{H}e^{\theta _{10,2}}&{}\frac{1}{H}e^{\theta _{10,3}}&{}\frac{1}{H}e^{\theta _{10,4}}&{}-\frac{1}{H}\lambda _{10}^2e^{\theta _{10,2}}&{}\omega _{10}\lambda _{10}e^{\theta _{10,1}}&{}...&{}\frac{1}{H}\lambda _{10}e^{\theta _{10,4}} \end{array}\right| ,\\ \Delta A^{(1)}_{41}=\left| \begin{array}{llllllllllll} 1&{}\omega _1e^{\theta _{1,1}}&{}\frac{1}{H}e^{\theta _{1,2}}&{}\frac{1}{H}e^{\theta _{1,3}}&{}\frac{1}{H}e^{\theta _{1,4}}&{}-\frac{1}{H}\lambda _1^2e^{\theta _{1,3}}&{}\omega _1\lambda _1e^{\theta _{1,1}}&{}...&{}\frac{1}{H}\lambda _1e^{\theta _{1,4}}\\ 1&{}\omega _2e^{\theta _{2,1}}&{}\frac{1}{H}e^{\theta _{2,2}}&{}\frac{1}{H}e^{\theta _{2,3}}&{}\frac{1}{H}e^{\theta _{2,4}}&{}-\frac{1}{H}\lambda _2^2e^{\theta _{2,3}}&{}\omega _2\lambda _2e^{\theta _{2,1}}&{}...&{}\frac{1}{H}\lambda _2e^{\theta _{2,4}}\\ ...&{}...&{}...&{}...&{}...&{}...&{}...&{}...&{}...\\ 1&{}\omega _{10}e^{\theta _{10,1}}&{}\frac{1}{H}e^{\theta _{10,2}}&{}\frac{1}{H}e^{\theta _{10,3}}&{}\frac{1}{H}e^{\theta _{10,4}}&{}-\frac{1}{H}\lambda _{10}^2e^{\theta _{10,3}}&{}\omega _{10}\lambda _{10}e^{\theta _{10,1}}&{}...&{}\frac{1}{H}\lambda _{10}e^{\theta _{10,4}} \end{array}\right| ,\\ \Delta A^{(1)}_{51}=\left| \begin{array}{llllllllllll} 1&{}\omega _1e^{\theta _{1,1}}&{}\frac{1}{H}e^{\theta _{1,2}}&{}\frac{1}{H}e^{\theta _{1,3}}&{}\frac{1}{H}e^{\theta _{1,4}}&{}-\frac{1}{H}\lambda _1^2e^{\theta _{1,4}}&{}\omega _1\lambda _1e^{\theta _{1,1}}&{}...&{}\frac{1}{H}\lambda _1e^{\theta _{1,4}}\\ 1&{}\omega _2e^{\theta _{2,1}}&{}\frac{1}{H}e^{\theta _{2,2}}&{}\frac{1}{H}e^{\theta _{2,3}}&{}\frac{1}{H}e^{\theta _{2,4}}&{}-\frac{1}{H}\lambda _2^2e^{\theta _{2,4}}&{}\omega _2\lambda _2e^{\theta _{2,1}}&{}...&{}\frac{1}{H}\lambda _2e^{\theta _{2,4}}\\ ...&{}...&{}...&{}...&{}...&{}...&{}...&{}...&{}...\\ 1&{}\omega _{10}e^{\theta _{10,1}}&{}\frac{1}{H}e^{\theta _{10,2}}&{}\frac{1}{H}e^{\theta _{10,3}}&{}\frac{1}{H}e^{\theta _{10,4}}&{}-\frac{1}{H}\lambda _{10}^2e^{\theta _{10,4}}&{}\omega _{10}\lambda _{10}e^{\theta _{10,1}}&{}...&{}\frac{1}{H}\lambda _{10}e^{\theta _{10,4}} \end{array}\right| ,\\ \end{array} \end{aligned}$$

where $\omega _j=\frac{K}{H}\left( \lambda _j+i\sqrt{1-\lambda ^2_j}\right)$, the *i* is an imaginary number, then $\theta _{j,1}=-2it+G_{(1,j)}$, $\theta _{j,i}=\left( -i\lambda _j-\sqrt{1-\lambda ^2_j}\right) x- \left( i\lambda ^2_j+i \right) t+G_{(1,j)}$, the ranges of the subscripts *j* and *i* of $\theta$ are $1\le i\le 4$, $1\le j\le 10$.

Based on the Eq. (49), we can obtain the following systems

59 $$\begin{aligned} \begin{array}{l} A^{(1)}_{21}=\frac{\Delta A^{(1)}_{21}}{\Delta _2}, A^{(1)}_{31}=\frac{\Delta A^{(1)}_{31}}{\Delta _2}, A^{(1)}_{41}=\frac{\Delta A^{(1)}_{41}}{\Delta _2}, A^{(1)}_{51}=\frac{\Delta A^{(1)}_{51}}{\Delta _2}, \end{array} \end{aligned}$$

the analytic 2-soliton solutions of four-component CNLS equations are obtained on zero and plane wave backgrounds by Darboux transformation as following

60 $$\begin{aligned} \begin{array}{l} \left\{ \begin{array}{lllllllll} {\widetilde{q}}_1=e^{-it}+2A^{(1)}_{21},\\ {\widetilde{q}}_2=2A^{(1)}_{31},\\ {\widetilde{q}}_3=2A^{(1)}_{41},\\ {\widetilde{q}}_4=2A^{(1)}_{51}. \end{array}\right. \end{array} \end{aligned}$$

We can obtain a new class of dark-bright-bright-bright soliton solutions(60), which admits the one-valley dark soliton in component $q_1$ and triple-hump bright solitons in the other three components.

#### N-soliton solutions on complex wave backgrounds

Next we make two non-zero seed solutions , so that we can get the novel N-soliton solutions on complex wave backgrounds. In order to obtain the *N*-soliton solution formula of the four-component coupled NLS equations on complex wave backgrounds with Darboux transformation, we give a set of seed solutions $q_1=q_2=e^{-2it}, q_3=q_4=0$ and substitute these solutions into Eqs. (4) and (5), which can get basic solutions for CNLS equations:

61 $$\begin{aligned} \begin{array}{l} \varphi =\left( \begin{array}{llllllll}Le^{\left( \sqrt{-\lambda ^2+2}\right) x+5it} \\ 0\\ 0\\ 0\\ 0\end{array}\right) , \phi =\left( \begin{array}{llllllll}0\\ e^{\left( \sqrt{-\lambda ^2+2}\right) x+it} \\ 0\\ 0\\ 0\end{array}\right) , \eta =\left( \begin{array}{llllllll}0\\ 0\\ e^{\left( \sqrt{-\lambda ^2+2}\right) x+it} \\ 0\\ 0\end{array}\right) ,\\ \xi =\left( \begin{array}{llllllll}0\\ 0\\ 0\\ e^{-i\lambda x-i\lambda ^2t} \\ 0\end{array}\right) , \varepsilon =\left( \begin{array}{llllllll}0\\ 0\\ 0\\ 0\\ e^{-i\lambda x-i\lambda ^2t}\end{array}\right) . \end{array} \end{aligned}$$

with $$L=\frac{4i\lambda }{(i\lambda -\sqrt{-\lambda ^2+2})(\lambda ^2-2)}$$.

Substituting Eq. (61) into Eq. (8), we give rise to

62 $$\begin{aligned} \begin{array}{l} M_j^{(1)}=\frac{1}{L}e^{-4it+G_{1,j}}, M_j^{(2)}=\frac{1}{L}e^{-4it+G_{2,j}},\\ M_j^{(3)}=\frac{1}{L}e^{\left( -i\lambda -\sqrt{-\lambda ^2+2}\right) x- \left( i\lambda ^2+5i \right) t+G_{3,j}}, M_j^{(4)}=\frac{1}{L}e^{\left( -i\lambda -\sqrt{-\lambda ^2+2}\right) x- \left( i\lambda ^2+5i \right) t+G_{4,j}}, \end{array} \end{aligned}$$

where $$e^{G_{1,j}}=v_j^{(11)}, e^{G_{2,j}}=v_j^{(22)}, e^{G_{3,j}}=v_j^{(33)}, e^{G_{4,j}}=v_j^{(44)}$$, $$1\le j\le 5N$$.

In order to obtain the N-soliton solution of Eq. (1) on complex wave backgrounds, we consider matrix *T* as follow:

63 $$\begin{aligned} \begin{array}{l} T=\left( \begin{array}{llllllll}\lambda ^{N}+\sum ^{N-1}_{i=0}A_{11}\lambda ^i_j&{}\sum ^{N-1}_{i=0}A_{12}\lambda ^i_j&{}\sum ^{N-1}_{i=0}A_{13}\lambda ^i_j&{}\sum ^{N-1}_{i=0}A_{14}\lambda ^i_j&{}\sum ^{N-1}_{i=0}A_{15}\lambda ^i_j\\ \sum ^{N-1}_{i=0}A_{21}\lambda ^i_j&{}\lambda ^{N}+\sum ^{N-1}_{i=0}A_{22}\lambda ^i_j&{}\sum ^{N-1}_{i=0}A_{23}\lambda ^i_j&{}\sum ^{N-1}_{i=0}A_{24}\lambda ^i_j&{}\sum ^{N-1}_{i=0}A_{25}\lambda ^i_j\\ \sum ^{N-1}_{i=0}A_{31}\lambda ^i_j&{}\sum ^{N-1}_{i=0}A_{32}\lambda ^i_j&{}\lambda ^{N}+\sum ^{N-1}_{i=0}A_{33}\lambda ^i_j&{}\sum ^{N-1}_{i=0}A_{34}\lambda ^i_j&{}\sum ^{N-1}_{i=0}A_{35}\lambda ^i_j\\ \sum ^{N-1}_{i=0}A_{41}\lambda ^i_j&{}\sum ^{N-1}_{i=0}A_{42}\lambda ^i_j&{}\sum ^{N-1}_{i=0}A_{43}\lambda ^i_j&{}\lambda ^{N}+\sum ^{N-1}_{i=0}A_{44\lambda ^i_j}&{}\sum ^{N-1}_{i=0}A_{45}\lambda ^i_j\\ \sum ^{N-1}_{i=0}A_{51}\lambda ^i_j&{}\sum ^{N-1}_{i=0}A_{52}\lambda ^i_j&{}\sum ^{N-1}_{i=0}A_{53}\lambda ^i_j&{}\sum ^{N-1}_{i=0}A_{54}\lambda ^i_j&{}\lambda ^{N}+\sum ^{N-1}_{i=0}A_{55}\lambda ^i_j \end{array}\right) , \end{array} \end{aligned}$$

and

64 $$\begin{aligned} \begin{array}{l} \left\{ \begin{array}{lllllllll} \sum _{i=0}^{N-1}(A_{11}^{(i)}+A_{12}^{(i)}M_j^{(1)}+A^{(i)}_{13}M^{(2)}_{j}+A^{(i)}_{14}M^{(3)}_{j}+A^{(i)}_{15}M^{(4)}_{j})\lambda _j^{i}=-\lambda _j^{N},\\ \sum _{i=0}^{N-1}(A_{21}^{(i)}+A_{22}^{(i)}M_j^{(1)}+A^{(i)}_{23}M^{(2)}_{j}+A^{(i)}_{24}M^{(3)}_{j}+A^{(i)}_{25}M^{(4)}_{j})\lambda _j^{i}=-M^{(1)}_j\lambda _j^{N},\\ \sum _{i=0}^{N-1}(A_{31}^{(i)}+A_{32}^{(i)}M_j^{(1)}+A^{(i)}_{33}M^{(2)}_{j}+A^{(i)}_{34}M^{(3)}_{j}+A^{(i)}_{35}M^{(4)}_{j})\lambda _j^{i}=-M^{(2)}_j\lambda _j^{N},\\ \sum _{i=0}^{N-1}(A_{41}^{(i)}+A_{42}^{(i)}M_j^{(1)}+A^{(i)}_{43}M^{(2)}_{j}+A^{(i)}_{44}M^{(3)}_{j}+A^{(i)}_{45}M^{(4)}_{j})\lambda _j^{i}=-M^{(3)}_j\lambda _j^{N},\\ \sum _{i=0}^{N-1}(A_{51}^{(i)}+A_{52}^{(i)}M_j^{(1)}+A^{(i)}_{53}M^{(2)}_{j}+A^{(i)}_{54}M^{(3)}_{j}+A^{(i)}_{55}M^{(4)}_{j})\lambda _j^{i}=-M^{(4)}_j\lambda _j^{N}. \end{array}\right. \end{array} \end{aligned}$$

The N-soliton solutions of Eq. (1) are obtained according to Cramer’s rule on complex wave backgrounds in Eq. (64) as following forms:

65 $$\begin{aligned} \begin{array}{l} \left\{ \begin{array}{lllllllll} {\widetilde{q}}_1=e^{-2it}+2A^{(N-1)}_{21},\\ {\widetilde{q}}_2=e^{-2it}+2A^{(N-1)}_{31},\\ {\widetilde{q}}_3=2A^{(N-1)}_{41},\\ {\widetilde{q}}_4=2A^{(N-1)}_{51}, \end{array}\right. \end{array} \end{aligned}$$

where

66 $$\begin{aligned} \begin{array}{l} A^{(N-1)}_{21}=\frac{\Delta A^{(N-1)}_{21}}{\Delta },~~ A^{(N-1)}_{31}=\frac{\Delta A^{(N-1)}_{31}}{\Delta },~~ A^{(N-1)}_{41}=\frac{\Delta A^{(N-1)}_{41}}{\Delta },~~ A^{(N-1)}_{51}=\frac{\Delta A^{(N-1)}_{51}}{\Delta }, \end{array} \end{aligned}$$

among them

67 $$\begin{aligned} \begin{array}{l} \Delta =\left| \begin{array}{lllllllllllllllll} 1&{}\frac{1}{L}_1e^{\theta _{1,1}}&{}...&{}\frac{1}{L}_1e^{\theta _{1,4}}&{}...&{}\lambda ^{(N-1)}_1&{}\frac{1}{L}_1\lambda ^{(N-1)}_1e^{\theta _{1,1}}&{}...&{}\frac{1}{L}_1\lambda ^{(N-1)}_1e^{\theta _{1,4}}&{}\\ 1&{}\frac{1}{L}_2e^{\theta _{2,1}}&{}...&{}\frac{1}{L}_2e^{\theta _{2,4}}&{}...&{}\lambda ^{(N-1)}_2&{}\frac{1}{L}_2\lambda ^{(N-1)}_2e^{\theta _{2,1}}&{}...&{}\frac{1}{L}_2\lambda ^{(N-1)}_2e^{\theta _{2,4}}&{}\\ ...&{}...&{}...&{}...&{}...&{}...&{}...&{}...&{}...\\ 1&{}\frac{1}{L}_{5N}e^{\theta _{5N,1}}&{}...&{}\frac{1}{L}_{5N}e^{\theta _{5N,4}}&{}...&{}\lambda ^{(N-1)}_{5N}&{}\frac{1}{L}_{5N}\lambda ^{(N-1)}_{5N}e^{\theta _{5N,1}}&{}...&{}\frac{1}{L}_{5N}\lambda ^{(N-1)}_{5N}e^{\theta _{5N,4}} \end{array}\right| ,\\ \Delta A^{(N-1)}_{21}=\left| \begin{array}{llllllllllllllllllc} 1&{}\frac{1}{L}_1e^{\theta _{1,1}}&{}\frac{1}{L}_1e^{\theta _{1,2}}&{}...&{}-\frac{1}{L}_1\lambda ^{(N)}_1e^{\theta _{1,1}}&{}\frac{1}{L}_1\lambda ^{(N-1)}_1e^{\theta _{1,1}}&{}...&{}\frac{1}{L}_1\lambda ^{(N-1)}_1e^{\theta _{1,4}}&{}\\ 1&{}\frac{1}{L}_2e^{\theta _{2,1}}&{}\frac{1}{L}_2e^{\theta _{2,2}}&{}...&{}-\frac{1}{L}_2\lambda ^{(N)}_2e^{\theta _{2,1}}&{}\frac{1}{L}_2\lambda ^{(N-1)}_2e^{\theta _{2,1}}&{}...&{}\frac{1}{L}_2\lambda ^{(N-1)}_2e^{\theta _{2,4}}&{}\\ ...&{}...&{}...&{}...&{}...&{}...&{}...&{}...\\ 1&{}\frac{1}{L}_{5N}e^{\theta _{5N,1}}&{}\frac{1}{L}_{5N}e^{\theta _{5N,2}}&{}...&{}-\frac{1}{L}_{5N}\lambda ^{(N)}_{5N}e^{\theta _{5N,1}}&{}\frac{1}{L}_{5N}\lambda ^{(N-1)}_{5N}e^{\theta _{5N,1}}&{}...&{}\frac{1}{L}_{5N}\lambda ^{(N-1)}_{5N}e^{\theta _{5N,4}} \end{array}\right| ,\\ \Delta A^{(N-1)}_{31}=\left| \begin{array}{lllllllllllllllllllllc} 1&{}\frac{1}{L}_1e^{\theta _{1,1}}&{}\frac{1}{L}_1e^{\theta _{1,2}}&{}...&{}-\frac{1}{L}_1\lambda ^{(N)}_1e^{\theta _{1,2}}&{}\frac{1}{L}_1\lambda ^{(N-1)}_1e^{\theta _{1,1}}&{}...&{}\frac{1}{L}_1\lambda ^{(N-1)}_1e^{\theta _{1,4}}&{}\\ 1&{}\frac{1}{L}_2e^{\theta _{2,1}}&{}\frac{1}{L}_2e^{\theta _{2,2}}&{}...&{}-\frac{1}{L}_2\lambda ^{(N)}_2e^{\theta _{2,2}}&{}\frac{1}{L}_2\lambda ^{(N-1)}_2e^{\theta _{2,1}}&{}...&{}\frac{1}{L}_2\lambda ^{(N-1)}_2e^{\theta _{2,4}}&{}\\ ...&{}...&{}...&{}...&{}...&{}...&{}...&{}...\\ 1&{}\frac{1}{L}_{5N}e^{\theta _{5N,1}}&{}\frac{1}{L}_{5N}e^{\theta _{5N,2}}&{}...&{}-\frac{1}{L}_{5N}\lambda ^{(N)}_{5N}e^{\theta _{5N,2}}&{}\frac{1}{L}_{5N}\lambda ^{(N-1)}_{5N}e^{\theta _{5N,1}}&{}...&{}\frac{1}{L}_{5N}\lambda ^{(N-1)}_{5N}e^{\theta _{5N,4}} \end{array}\right| ,\\ \Delta A^{(N-1)}_{41}=\left| \begin{array}{lllllllllllllllllllllc} 1&{}\frac{1}{L}_1e^{\theta _{1,1}}&{}\frac{1}{L}_1e^{\theta _{1,2}}&{}...&{}-\frac{1}{L}_1\lambda ^{(N)}_1e^{\theta _{1,3}}&{}\frac{1}{L}_1\lambda ^{(N-1)}_1e^{\theta _{1,1}}&{}...&{}\frac{1}{L}_1\lambda ^{(N-1)}_1e^{\theta _{1,4}}&{}\\ 1&{}\frac{1}{L}_1e^{\theta _{2,1}}&{}\frac{1}{L}_2e^{\theta _{2,2}}&{}...&{}-\frac{1}{L}_2\lambda ^{(N)}_2e^{\theta _{2,3}}&{}\frac{1}{L}_2\lambda ^{(N-1)}_2e^{\theta _{2,1}}&{}...&{}\frac{1}{L}_2\lambda ^{(N-1)}_2e^{\theta _{2,4}}&{}\\ ...&{}...&{}...&{}...&{}...&{}...&{}...&{}...\\ 1&{}\frac{1}{L}_1e^{\theta _{5N,1}}&{}\frac{1}{L}_{5N}e^{\theta _{5N,2}}&{}...&{}-\frac{1}{L}_{5N}\lambda ^{(N)}_{5N}e^{\theta _{5N,3}}&{}\frac{1}{L}_{5N}\lambda ^{(N-1)}_{5N}e^{\theta _{5N,1}}&{}...&{}\frac{1}{L}_{5N}\lambda ^{(N-1)}_{5N}e^{\theta _{5N,4}} \end{array}\right| ,\\ \Delta A^{(N-1)}_{51}=\left| \begin{array}{llllllllllllllllllc} 1&{}\frac{1}{L}_1e^{\theta _{1,1}}&{}\frac{1}{L}_1e^{\theta _{1,2}}&{}...&{}-\frac{1}{L}_1\lambda ^{(N)}_1e^{\theta _{1,4}}&{}\frac{1}{L}_1\lambda ^{(N-1)}_1e^{\theta _{1,1}}&{}...&{}\frac{1}{L}_1\lambda ^{(N-1)}_1e^{\theta _{1,4}}&{}\\ 1&{}\frac{1}{L}_2e^{\theta _{2,1}}&{}\frac{1}{L}_2e^{\theta _{2,2}}&{}...&{}-\frac{1}{L}_2\lambda ^{(N)}_2e^{\theta _{2,4}}&{}\frac{1}{L}_2\lambda ^{(N-1)}_2e^{\theta _{2,1}}&{}...&{}\frac{1}{L}_2\lambda ^{(N-1)}_2e^{\theta _{2,4}}&{}\\ ...&{}...&{}...&{}...&{}...&{}...&{}...&{}...\\ 1&{}\frac{1}{L}_{5N}e^{\theta _{5N,1}}&{}\frac{1}{L}_{5N}e^{\theta _{5N,2}}&{}...&{}-\frac{1}{L}_{5N}\lambda ^{(N)}_{5N}e^{\theta _{5N,4}}&{}\frac{1}{L}_{5N}\lambda ^{(N-1)}_{5N}e^{\theta _{5N,1}}&{}...&{}\frac{1}{L}_{5N}\lambda ^{(N-1)}_{5N}e^{\theta _{5N,4}} \end{array}\right| , \end{array} \end{aligned}$$

with $$L_j=\frac{4i\lambda _j}{\left( i\lambda _j-\sqrt{-\lambda _j^2+2}\right) \left( \lambda _j^2-2 \right) }$$, $$\theta _{1,1}=-4it+G_{(1,1)}$$, $$\theta _{1,2}=-4it+G_{(2,1)}$$, $$\theta _{1,4}=\left( -i\lambda -\sqrt{-\lambda ^2+2}\right) x- \left( i\lambda ^2+5i \right) t+G_{4,1}$$, $$\theta _{2,1}=-4it+G_{(1,2)}$$, $$\theta _{2,2}=-4it+G_{(2,2)}$$, $$\theta _{2,4}=\left( -i\lambda -\sqrt{-\lambda ^2+2}\right) x- \left( i\lambda ^2+5i \right) t+G_{4,2}$$, $$\theta _{5N,1}=-4it+G_{(1,5N)}$$,$$\theta _{5N,2}=-4it+G_{(2,5N)}$$, $$\theta _{5N,4}=\left( -i\lambda -\sqrt{-\lambda ^2+2}\right) x- \left( i\lambda ^2+5i \right) t+G_{4,5N}$$.

In order to obtain 1-soliton solutions of the four-component coupled NLS equations on complex wave backgrounds, we consider $N=1$ in Eqs. (63) and (64), and obtain the matrix *T*

68 $$\begin{aligned} \begin{array}{l} T=\left( \begin{array}{llllllll} \lambda +A^{(0)}_{11}&{}A^{(0)}_{12}&{}A^{(0)}_{13}&{}A^{(0)}_{14}&{}A^{(0)}_{15}\\ A^{(0)}_{21}&{}\lambda +A^{(0)}_{22}&{}A^{(0)}_{23}&{}A^{(0)}_{24}&{}A^{(0)}_{25}\\ A^{(0)}_{31}&{}A^{(0)}_{32}&{}\lambda +A^{(0)}_{33}&{}A^{(0)}_{34}&{}A^{(0)}_{35}\\ A^{(0)}_{41}&{}A^{(0)}_{42}&{}A^{(0)}_{43}&{}\lambda +A^{(0)}_{44}&{}A^{(0)}_{45}\\ A^{(0)}_{51}&{}A^{(0)}_{52}&{}A^{(0)}_{53}&{}A^{(0)}_{54}&{}\lambda +A^{(0)}_{55}\end{array}\right) , \end{array} \end{aligned}$$

and

69 $$\begin{aligned} \begin{array}{l} \left\{ \begin{array}{lllllllll} A^{(0)}_{11}+A^{(0)}_{12}M_{j}^{(1)}+A^{(0)}_{13}M_{j}^{(2)}+A^{(0)}_{14}M_{j}^{(3)}+A^{(0)}_{15}M_{j}^{(4)}=-\lambda _j,\\ A^{(0)}_{21}+A^{(0)}_{22}M_{j}^{(1)}+A^{(0)}_{23}M_{j}^{(2)}+A^{(0)}_{24}M_{j}^{(3)}+A^{(0)}_{25}M_{j}^{(4)}=-M^{(1)}_j\lambda _j,\\ A^{(0)}_{31}+A^{(0)}_{32}M_{j}^{(1)}+A^{(0)}_{33}M_{j}^{(2)}+A^{(0)}_{34}M_{j}^{(3)}+A^{(0)}_{35}M_{j}^{(4)}=-M^{(2)}_j\lambda _j,\\ A^{(0)}_{41}+A^{(0)}_{42}M_{j}^{(1)}+A^{(0)}_{43}M_{j}^{(2)}+A^{(0)}_{44}M_{j}^{(3)}+A^{(0)}_{45}M_{j}^{(4)}=-M^{(3)}_j\lambda _j,\\ A^{(0)}_{51}+A^{(0)}_{52}M_{j}^{(1)}+A^{(0)}_{53}M_{j}^{(2)}+A^{(0)}_{54}M_{j}^{(3)}+A^{(0)}_{55}M_{j}^{(4)}=-M^{(4)}_j\lambda _j. \end{array}\right. \end{array} \end{aligned}$$

According to Eq. (69) and Cramer’s rule, we can derive the systems:

$$\begin{aligned} \Delta _1=\left| \begin{array}{llllllllllllll} 1&{}\frac{1}{L}_1e^{\theta _{1,1}}&{}\frac{1}{L}_1e^{\theta _{1,2}}&{}\frac{1}{L}_1e^{\theta _{1,3}}&{}\frac{1}{L}_1e^{\theta _{1,4}}\\ 1&{}\frac{1}{L}_2e^{\theta _{2,1}}&{}\frac{1}{L}_2e^{\theta _{2,2}}&{}\frac{1}{L}_2e^{\theta _{2,3}}&{}\frac{1}{L}_2e^{\theta _{2,4}}\\ ...&{}...&{}...&{}...&{}...\\ ...&{}...&{}...&{}...&{}...\\ 1&{}\frac{1}{L}_5e^{\theta _{5,1}}&{}\frac{1}{L}_5e^{\theta _{5,2}}&{}\frac{1}{L}_5e^{\theta _{5,3}}&{}\frac{1}{L}_5e^{\theta _{5,4}} \end{array}\right| , \\ \Delta A^{(0)}_{21}=\left| \begin{array}{lllllllllllllll} -\frac{1}{L}_1\lambda _1e^{\theta _{1,1}}&{}\frac{1}{L}_1e^{\theta _{1,1}}&{}\frac{1}{L}_1e^{\theta _{1,2}}&{}\frac{1}{L}_1e^{\theta _{1,3}}&{}\frac{1}{L}_1e^{\theta _{1,4}}\\ -\frac{1}{L}_2\lambda _2e^{\theta _{2,1}}&{}\frac{1}{L}_2e^{\theta _{2,1}}&{}\frac{1}{L}_2e^{\theta _{2,2}}&{}\frac{1}{L}_2e^{\theta _{2,3}}&{}\frac{1}{L}_2e^{\theta _{2,4}}\\ ...&{}...&{}...&{}...&{}...\\ ...&{}...&{}...&{}...&{}...\\ -\frac{1}{L}_5\lambda _5e^{\theta _{5,1}}&{}\frac{1}{L}_5e^{\theta _{5,1}}&{}\frac{1}{L}_5e^{\theta _{5,2}}&{}\frac{1}{L}_5e^{\theta _{5,3}}&{}\frac{1}{L}_5e^{\theta _{5,4}} \end{array}\right| , \\ \Delta A^{(0)}_{31}=\left| \begin{array}{lllllllllllllllc} -\frac{1}{L}_1\lambda e^{\theta _{1,2}}&{}\frac{1}{L}_1e^{\theta _{1,1}}&{}\frac{1}{L}_1e^{\theta _{1,2}}&{}\frac{1}{L}_1e^{\theta _{1,3}}&{}\frac{1}{L}_1e^{\theta _{1,4}}\\ -\frac{1}{L}_2\lambda e^{\theta _{2,2}}&{}\frac{1}{L}_2e^{\theta _{2,1}}&{}\frac{1}{L}_2e^{\theta _{2,2}}&{}\frac{1}{L}_2e^{\theta _{2,3}}&{}\frac{1}{L}_2e^{\theta _{2,4}}\\ ...&{}...&{}...&{}...&{}...\\ ...&{}...&{}...&{}...&{}...\\ -\frac{1}{L}_5\lambda e^{\theta _{5,2}}&{}\frac{1}{L}_5e^{\theta _{5,1}}&{}\frac{1}{L}_5e^{\theta _{5,2}}&{}\frac{1}{L}_5e^{\theta _{5,3}}&{}\frac{1}{L}_5e^{\theta _{5,4}} \end{array}\right| , \\ \Delta A^{(0)}_{41}= \left| \begin{array}{lllllllllllllllll} -\frac{1}{L}_1\lambda _1e^{\theta _{1,3}}&{}\frac{1}{L}_1e^{\theta _{1,1}}&{}\frac{1}{L}_1e^{\theta _{1,2}}&{}\frac{1}{L}_1e^{\theta _{1,3}}&{}\frac{1}{L}_1e^{\theta _{1,4}}\\ -\frac{1}{L}_2\lambda _2e^{\theta _{2,3}}&{}\frac{1}{L}_2e^{\theta _{2,1}}&{}\frac{1}{L}_2e^{\theta _{2,2}}&{}\frac{1}{L}_2e^{\theta _{2,3}}&{}\frac{1}{L}_2e^{\theta _{2,4}}\\ ...&{}...&{}...&{}...&{}...\\ ...&{}...&{}...&{}...&{}...\\ -\frac{1}{L}_5\lambda _5e^{\theta _{5,3}}&{}\frac{1}{L}_5e^{\theta _{5,1}}&{}\frac{1}{L}_5e^{\theta _{5,2}}&{}\frac{1}{L}_5e^{\theta _{5,3}}&{}\frac{1}{L}_5e^{\theta _{5,4}} \end{array}\right| , \end{aligned}$$

70 $$\begin{aligned} \begin{array}{l} \Delta A^{(0)}_{51}=\left| \begin{array}{lllllllllllllllll} -\frac{1}{L}_1\lambda _1e^{\theta _{1,4}}&{}\frac{1}{L}_1e^{\theta _{1,1}}&{}\frac{1}{L}_1e^{\theta _{1,2}}&{}\frac{1}{L}_1e^{\theta _{1,3}}&{}\frac{1}{L}_1e^{\theta _{1,4}}\\ -\frac{1}{L}_2\lambda _2e^{\theta _{2,4}}&{}\frac{1}{L}_2e^{\theta _{2,1}}&{}\frac{1}{L}_2e^{\theta _{2,2}}&{}\frac{1}{L}_2e^{\theta _{2,3}}&{}\frac{1}{L}_2e^{\theta _{2,4}}\\ ...&{}...&{}...&{}...&{}...\\ ...&{}...&{}...&{}...&{}...\\ -\frac{1}{L}_5\lambda _5e^{\theta _{5,4}}&{}\frac{1}{L}_5e^{\theta _{5,1}}&{}\frac{1}{L}_5e^{\theta _{5,2}}&{}\frac{1}{L}_5e^{\theta _{5,3}}&{}\frac{1}{L}_5e^{\theta _{5,4}} \end{array}\right| .\\ \end{array} \end{aligned}$$

with $$L_j=\frac{4i\lambda _j}{\left( i\lambda _j-\sqrt{-\lambda _j^2+2}\right) \left( \lambda _j^2-2 \right) }$$, $$\theta _{1,1}=-4it+G_{(1,1)}$$, $$\theta _{1,2}=-4it+G_{(2,1)}$$, $$\theta _{1,3}=\left( -i\lambda -\sqrt{-\lambda ^2+2}\right) x- \left( i\lambda ^2+5i \right) t+G_{3,1},$$ $$\theta _{1,4}=\left( -i\lambda -\sqrt{-\lambda ^2+2}\right) x-\left( i\lambda ^2+5i \right) t+G_{4,1}$$, $$\theta _{2,1}=-4it+G_{(1,2)}$$, $$\theta _{2,2}=-4it+G_{(2,2)}$$, $$\theta _{2,3}=\left( -i\lambda -\sqrt{-\lambda ^2+2}\right) x- \left( i\lambda ^2+5i \right) t+G_{3,2},$$ $$\theta _{2,4}=\left( -i\lambda -\sqrt{-\lambda ^2+2}\right) x- \left( i\lambda ^2+5i \right) t+G_{4,2}$$, $$\theta _{5,1}=-4it+G_{(1,5)}$$, $$\theta _{5,2}=-4it+G_{(2,5)}$$, $$\theta _{5,3}=\left( -i\lambda -\sqrt{-\lambda ^2+2}\right) x-\left( i\lambda ^2+5i \right) t+G_{3,5},$$ $$\theta _{5,4}=\left( -i\lambda -\sqrt{-\lambda ^2+2}\right) x-\left( i\lambda ^2+5i \right) t+G_{4,5}$$.

Based on Eq. (70), we can obtain the following systems

71 $$\begin{aligned} \begin{array}{l} A^{(0)}_{21}=\frac{\Delta A^{(0)}_{21}}{\Delta _1},~~ A^{(0)}_{31}=\frac{\Delta A^{(0)}_{31}}{\Delta _1},~~ A^{(0)}_{41}=\frac{\Delta A^{(0)}_{41}}{\Delta _1},~~ A^{(0)}_{51}=\frac{\Delta A^{(0)}_{51}}{\Delta _1}, \end{array} \end{aligned}$$

the analytic 1-soliton solutions of four-component CNLS equations on complex wave backgrounds are obtained by Darboux transformation as following

72 $$\begin{aligned} \begin{array}{l} \left\{ \begin{array}{lllllllll} {\widetilde{q}}_1=e^{-2it}+2A^{(0)}_{21},\\ {\widetilde{q}}_2=e^{-2it}+2A^{(0)}_{31},\\ {\widetilde{q}}_3=2A^{(0)}_{41},\\ {\widetilde{q}}_4=2A^{(0)}_{51}. \end{array}\right. \end{array} \end{aligned}$$

The collision properties between dark-dark-bright-bright solitons (72) are considered, and the vector solitons are expected to be much more abundant than those of previously reported vector soliton collisions. Some soliton solutions and interactions of four-component CNLS equations on complex wave backgrounds are considered, which can describe the four-component BECs. Furthermore, some dark soliton solutions can exist widely in the collision processes. We investigate the wave structures of four-component NLS equations with mixed nonzero and zero wave conditions, which can be used to describe the interactions of many bodies in differential fields. And, we find some dark soliton solutions (non-zero backgrounds) and soliton-like solutions (zero backgrounds) with the aid of the Darboux transformation.

## Data Availability

All data generated or analysed during this study are included in this published article.
